# Diversity and potential activity patterns of planktonic eukaryotic microbes in a mesoeutrophic coastal area (eastern English Channel)

**DOI:** 10.1371/journal.pone.0196987

**Published:** 2018-05-10

**Authors:** Sara Rachik, Urania Christaki, Luen Luen Li, Savvas Genitsaris, Elsa Breton, Sébastien Monchy

**Affiliations:** 1 Univ. Littoral Côte d’Opale, CNRS, Univ. Lille, UMR, LOG, Laboratoire d'Océanologie et de Géosciences, Lille, France; 2 International Hellenic University, School of Economics, Business Administration & Legal Studies, Thessaloniki, Greece; Wilfrid Laurier University, CANADA

## Abstract

The diversity of planktonic eukaryotic microbes was studied at a coastal station of the eastern English Channel (EEC) from March 2011 to July 2015 (77 samples) using high throughput sequencing (454-pyrosequencing and Illumina) of the V2-V3 hypervariable region of the 18S SSU rDNA gene. Similar estimations of OTU relative abundance and taxonomic distribution for the dominant higher taxonomic groups (contributing >1% of the total number of OTUs) were observed with the two methods (Kolmogorov-Smirnov p-value = 0.22). Eight super-groups were identified throughout all samples: Alveolata, Stramenopiles, Opisthokonta, Hacrobia, Archeaplastida, Apusozoa, Rhizaria, and Amoebozoa (ordered by decreasing OTU richness). To gain further insight into microbial activity in the EEC, ribosomal RNA was extracted for samples from 2013–2015 (30 samples). Analysis of 18S rDNA and rRNA sequences led to the detection of 696 and 700 OTUs, respectively. Cluster analysis based on OTUs’ abundance indicated three major seasonal groups that were associated to spring, winter/autumn, and summer conditions. The clusters inferred from rRNA data showed a clearer seasonal representation of the community succession than the one based on rDNA. The rRNA/rDNA ratio was used as a proxy for relative cell activity. When all OTUs were considered, the average rRNA:rDNA ratio showed a linear trend around the 1:1 line, suggesting a linear relation between OTU abundance (rDNA) and activity (rRNA). However, this ratio was highly variable over time when considering individual OTUs. Interestingly, the OTU affiliated with *P*. *globosa* displayed rRNA:rDNA ratio that allowed to delimit high *vs* low abundance and high *vs* low activity periods. It unveiled quite well the *Phaeocystis* bloom dynamic regarding cell proliferation and activity, and could even be used as early indicator of an upcoming bloom.

## Introduction

Planktonic eukaryote microbes are abundant, ubiquitous in aquatic environments and extremely diverse in terms of taxonomy and metabolism [1]. They play a crucial role in the functioning of marine ecosystems through primary production (photosynthetic phytoplankton), predation (heterotrophs and mixotrophs) and/or symbiosis (mutualists and parasites) [1,2]. Studying their diversity and metabolic activity is, therefore, fundamental for understanding the functioning of marine ecosystems [3,4,5,6]. High-throughput sequencing targeting the 16S/18S rDNA genes coupled with analysis using efficient bioinformatics tools, made it possible to reveal the vast diversity of marine microbes. In marine ecosystems, this approach has been used to explore spatial and temporal prokaryote and eukaryote diversity in different regions worldwide [7,8,9,10,11,12] and revealed the diversity overlooked by conventional methods (e.g microscopy) [11,13,14]. Sequencing of rDNA allows detecting a large number of microorganisms despite their viability and activity. However, estimating microbial activity is fundamental to understanding the functioning of ecosystems. Generally, the quantity of rRNA is proportional to both the number of ribosomes and total RNA concentration [15,16], and can therefore be used as an indicator of cell activity which cannot be assessed targeting only rDNA. Several studies have applied this strategy to marine microbial communities of bacteria [17,18,19,20], archaea [21], and eukaryotes [10,12,22,23,24,25,26]. The approach is based on the calculation of rRNA:rDNA ratio in order to normalize the rRNA concentration according to the number of cells (DNA concentration), as rDNA is more constant per cell [15,27,28]. However, due to variations depending on microbial life strategies, life histories and non-growth activities, limitations exist for using this ratio to infer cells activity [29]. Rather, such ratio may potentially infer species-specific life-cycle events [29]. While favorable environmental conditions are known triggers for increased cell activities, several studies showed that gene regulatory networks governing microbial life strategy is more complex than initially thought. Indeed, microorganisms experiencing repeated patterns of changing environmental conditions develop “anticipatory life strategies” based on adaptive regulatory gene networks [30,31,32]. Cells use past conditions as predictive signals to anticipate upcoming conditions. For example, they accumulate rRNA during unfavorable conditions, in period of low activity, shortly before favorable conditions return [33,34]. To our knowledge, such “anticipatory life strategy” has not been reported for eukaryote microbes in marine environments.

The present study was carried out in the eastern English Channel (EEC) in the framework of the SOMLIT network (French Network of Coastal Observatories). EEC is a meso-eutrophic coastal ecosystem presenting great seasonal fluctuations both in its biotic and abiotic environment. This ecosystem is characterized by recurrent massive blooms of *P*. *globosa* that develop under nitrogen-replete conditions and a silicate limitation at the end of winter [35,36,37], associated with sufficient underwater light intensity in order to achieve high growth rates [35,38]. *Phaeocystis* spp. exhibits alternate life cycles between solitary cells and gelatinous colonies [39], its bloom is preceded and followed by communities of colonial diatoms and dinoflagellate grazers (e.g [40,41,42,43] and references therein). In the EEC, the structure and seasonal succession of microbial eukaryotes community was thoroughly investigated through DNA-based high throughput sequencing [44,45,46]. However, information on the seasonal dynamic of relative cell activity is still lacking.

Our main objectives were to establish an overview of diversity in the mesoeutrophic coastal area of EEC and to study relationships between diversity and potential activity in microbial eukaryote communities by establishing rRNA:rDNA ratios. The study was conducted over 4 years at a coastal station of EEC from March 2011 to July 2015, using high-throughput sequencing. Specifically, the main questions were as follows: (i) what are the composition and succession of planktonic eukaryote communities in relation to environmental parameters in the EEC? (ii) What are the main differences/similarities between the rDNA and rRNA datasets? (iii) What is the ecological relevance of the rDNA:rRNA ratio values?

## Materials and methods

### Sampling area

**Fig 1:**
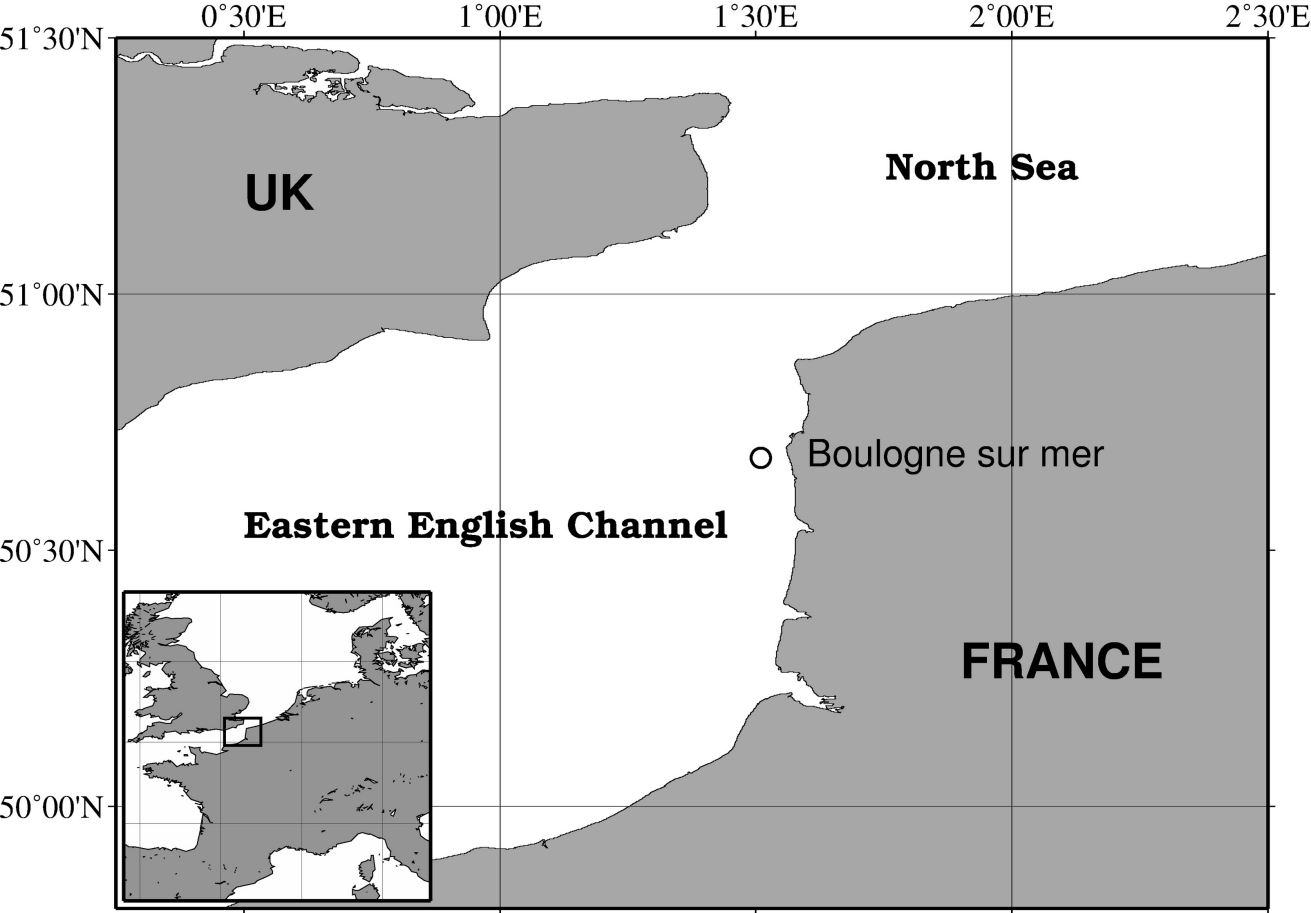
Study area in the eastern English Channel. The dot (●) indicates the location of the sampling station.

The sampling was carried out at the SOMLIT station (French Network of Coastal Observatories; http://somlit.epoc.u-bordeaux1.fr/fr/) in the EEC, one mile from the coast (50° 40’ 75” N, 1° 31’ 17” E; 20–25 maximum depth, Fig 1). For diversity assessment, subsurface samples (2–3 m water depth) were collected in 5 L polyethylene bottles, stored in the dark at *in situ* surface temperature, and filtered within 2h. Before filtration, the seawater sampled was prefiltered using a 150 μm mesh, in order to remove metazoa and large particles. Filtration, through 0.2 μm nucleopore filters (47 mm diameter), was performed using a very low filtration pressure peristaltic pump (15 rpm) in order to avoid filter clumping and minimize organism disruption. The filters were immediately stored at -80°C until DNA and RNA extraction. A total of 77 samples were collected between March 2011 and July 2015 (12, 20, 25, 12 and 8 samples for each year, respectively).

### Environmental variables

Sampling was performed using Niskin bottles coupled to a CTD Seabird (SBE 19) for measuring Sea Surface Temperature (SST, °C), salinity (S), and the diffuse attenuation coefficient for down-welling irradiance (*K*_*d*_, m^−1^), using a biospherical PAR light sensor (QSP 2300, Biospherical Instruments) connected to the CTD. The average sub-surface daily light intensity (I, E m^-2^ d^-1^) reaching phytoplankton was estimated using the formula (1) based on [47], where, *Z* was the depth at which samples were collected, and *I*_*0*_ was the daily incident light estimated from global solar radiation (GSR, W m^−2^). *I*_*0*_ was measured continuously every 5 min with a solar radiation sensor (Vantage Pro, Davis).

$$I=\frac{I_{0}(1-e^{-k_{d}Z})}{k_{d}Z}$$ (1)

Oxygen concentrations (ml L^-1^) were measured in triplicate by Winkler microtitration [48]. The pH was determined with a pH1970i (WTW) pH meter. Nutrient samples (Nitrate NO_3_^-^, Nitrite NO_2_^-^, Phosphate PO_4_^3-^ and Silicate SiOH_4_, μM) were analyzed on Alliance Integral Futura Autoanalyzer II according to Aminot and Kerouel (2004). Suspended Particulate Matter (SPM, mg L^-1^) was determined by weighing before and after filtration through GF/F filters (0.7μm) (for more details: http://somlit.epoc.u-bordeaux1.fr/fr/). Particulate Organic Carbon and Nitrogen (POC and PON, μg L^-1^) were estimated using a NA2100 Frisons Analyzer. Chlorophyll a (Chl. a, μg L^-1^) samples were extracted for 24h in 90% acetone following the fluorimetric protocol of Aminot and Kerouel (2004). The concentrations were measured using a 10-AU Turner Designs® fluorometer and the Lorenzen equations (1967).

### DNA and RNA extraction

Total DNA and RNA of planktonic microorganisms were extracted and purified simultaneously from the same filter with the Qiagen AllPrep DNA/RNA Mini kit (Qiagen, Hilden, Germany) following the manufacturer’s protocol. To remove contaminating DNA from RNA and inversely, all RNA samples were treated with DNase and all DNA samples were treated with RNase. Each resulting extraction (100μL) had DNA concentration between 0.26 and 28 ng μL^-1^ and RNA concentration between 1.14 and 14.8 ng μL^-1^, as measured with the Qubit® 2.0 fluorometer (Thermo Fisher Scientific, Waltham, MA, USA).

### PCR and tag Illumina sequencing

Extracted RNA was first denatured in presence of two primers, 18S-82F (5’-GAAACTGCGAATGGCTC-3’, [49]) and Euk-516r (5’-ACCAGACTTGCCCTCC-3’, [50]), that were designed to amplify the variable V2-V3 eukaryotic 18S rRNA gene regions (around 470–480 bp), at 70°C for 5 min and 25°C for 10 min. Then, it was reverse transcribed into cDNA using M-MLV reverse transcriptase RNAse H Minus DNA Polymerase (Euromedex, Souffelweyersheim, France) at 45°C for 60 min then held at 10°C. Afterwards, cDNA samples were treated with RNAse A (Euromedex, Souffelweyersheim, France) at 37°C for 10 min and were purified by using NucleoFast 96 PCR Clean-up Kit (Macherey Nagel, Düren, Germany). For amplicon library constructions, DNA and cDNA samples were first PCR amplified with primers Euk-82F and Euk-516R, and resulting products were subsequently attached to sequencing adapters-index through the second PCR step (Pegase platform, GenesDiffusion, Lille, France). The Platinium *Taq* High-Fidelity DNA Polymerase (Thermo Fisher Scientific, Waltham, MA, USA) was used for PCR amplifications with program set as: 94°C for 1 min, [15 s at 94°C, 15s at 51°C, 45 s at 68°C](26 cycles for 1st PCR, 9 cycles for 2nd PCR), and 1 min at 68°C. Products of second PCR were purified with the Agencourt AMPure XP system (Beckman Coulter, Brea, CA, USA), quantified with the PicoGreen Assay (Thermo Fisher Scientific, Waltham, MA, USA), and examined for quality with Bioanalyzer High sensitivity DNA Analysis Kits (Agilent, Santa Clara, CA, USA). Amplicon libraries were sequenced with the Illumina MiSeq platform 2x300bp paired-end run at the Plateforme de Génomique LIGAN-PM (Université de Lille 2, CNRS-UMR8199, Lille, France).

### Sequences processing

The rDNA and rRNA sequences obtained from Illumina were all processed together using the MOTHUR v1.34.0 software [51] following the standard operating procedure (http://www.mothur.org/wiki/MiSeq_SOP) [52]. Sequences were extracted and separated according to their index tag. The dataset was dereplicated to unique sequences and aligned against the SILVA 108 database (http://www.arb-silva.de/). Suspected chimeras were removed by using the UCHIME software [53]. After quality filtering, an average of 19,878 rDNA reads and 10,091 rRNA reads per sample were clustered into operational taxonomical units (OTUs) at 97% similarity threshold [5], using the average neighbor method in Mothur. Single singleton, referring to OTU that has a single representative sequence in the whole data set, were removed as these are most likely erroneous sequencing products [5,54]. After normalization of the entire dataset, all remaining OTUs (933 OTUs) sequences were searched against the PR2 curated database [55] and SILVA 114 database [56] by using BLASTN [57]. Careful examination and manual curation of BLASTN results was done to assign putative taxonomic affiliations for each OTU. Those identified as metazoa were removed from analyses to only target microbial eukaryotes.

### Data analyses

In order to establish an overview of microbial diversity over four years, we combined data from February 2013 to July 2015 (this study, GenBank-SRA accession SRP136006) with data previously obtained by our group from March 2011 to July 2013 (GenBank-SRA accession SRX768577, [45]). To note, the same set of primers (i.e. Euk-82F and Euk-516R) were used in both surveys for the amplification of 18S ribosomal gene. The two datasets were analyzed independently, and obtained results were compared for OTUs richness and succession.

Alpha diversity estimators (the richness estimator Chao-1, Simpson and Equitability indices) were calculated using the Past 3.05 software [58] for all samples. The Chao-1 estimator uses the number of singletons and doubletons to estimate the theoretically number of expected OTUs based on the singletons and doubletons in each sample. Simpson’s index (D) measures the probability that two individuals belong to the same OTU, it ranges from 0 (all taxa are equally present) to 1 (one taxon dominates in the community). The Shannon equitability index measuring the evenness of the community was calculated by dividing the Shannon diversity index (H') by the maximum diversity (H’max). These diversity estimators were compared for each date with a 'bootstrapping randomization procedure' and the *p*-value was computed based on 1,000 random permutation pairs.

Principal Component Analysis (PCA) was performed on environmental variables with R (v 3.2.3) [59] using the 'ade4' package [60]. Before PCA analysis, the BoxCox transformation [61] was applied using the 'caret' package [62] to approach normal distribution in the data. Sampling dates were grouped using the hierarchical Ward's method [63].

Microbial assemblages (based on OTUs) were grouped across sampling dates by hierarchical cluster analysis using the PRIMER version 6.0 [64]. The Bray-Curtis dissimilarity coefficients were calculated to build a matrix based on OTUs abundance. Similarity profile test (SIMPROF) was performed to define significant seasonal clusters [64].

The relative read abundance of each OTU was calculated at each date by dividing its number of reads (rRNA or rDNA) by the total reads number (rRNA or rDNA) for that OTU. For each OTU, the relative abundance of rRNA (y-axis) and rDNA (x-axis) reads per date were plotted on logarithmic scales and linear regression was calculated. This regression, used to estimate the relation between OTU abundance (rDNA) and relative activity (rRNA), was calculated using the Past 3.05 software [58], with RMA (Reduced Major Axis) to minimize both the x and y errors. For each OTU and date, rRNA:rDNA ratio was calculated by dividing the relative abundance of rRNA reads for an OTU by the relative abundance of rDNA reads of the same OTU. OTUs occurring only in the DNA or only in the RNA dataset were excluded for calculation of rRNA:rDNA ratio and for the linear regression analysis.

## Results

### Environmental variables and biological seasonality

During the study period, seawater temperature (SST) and salinity (S) ranged from 5.4 to 18.5°C and from 33.11 to 34.07, respectively (Table 1). Each year, the largest amounts of nutrients (26.7 μM for NO_3_^-^+NO_2_^-^, 1.2 μM for PO_4_^3-^, and 13.6 μM for SiOH_4_) were observed between November and March (Table 1). The N/P ratio dropped during *P*. *globosa* blooms (as low as 0.19 mol:mol in April 2012, S1 Table). Highest O_2_ concentrations were recorded every year during the phytoplankton spring bloom (up to 8.2 ml.L^-1^ in April of most years) and then decreased from May to September (down to 4.9 ml.L^-1^ in September 2014) (S1 Table). Chl. a amounts varied from 0.4 to 14.2 μg.L^-1^ with recurrent maximum values between March and June. The highest values of SPM were recorded in winter every year, except for June 2015 where it reached its maximum (32.9 mg.L^-1^) (S1 Table).

**Table 1:** List of environmental variables, means (±SD) of all measurements, minimal and maximal value measured per variable at the SOMLIT station (mean±sd, min-max).

	mean±sd	min-max
Kd (m^-1^)	0.37±0.1	0.17–0.73
PAR (E m^-2^ d^-1^)	22.35±13.5	2.28–51.95
T (°C)	12.07±3.9	5.44–18.5
S	34.11±0.3	33.11–34.7
O_2_ (ml.L^-1^)	6.42±0.9	4.9–8.2
pH	8.09±1.1	8.01–8.49
NO_3_^-^+NO_2_^-^ (μM)	5.89±7.3	0.06–26.71
PO_4_^3-^ (μM)	0.3±0.2	0.01–1.2
SiOH_4_ (μM)	2.57±3.4	0.01–13.6
POC (μgC.L^-1^)	367.03±283.5	64.78–1242.8
PON (μgN.L^-1^)	55.11±29.68	5.33–180.53
SPM (mg.L^-1^)	5.18±6.1	0.1–32.93
Chl. a (μg.L^-1^)	4.03±3.3	0.4–14.19
N/P	29.9±57.8	0.12–373.5

Kd: diffuse attenuation coefficient, PAR: photosynthetically active radiation, T: temperature, S: salinity, O_2_: Oxygen, pH: potential hydrogen, NO_3_^-^: nitrate, NO_2_^-^: nitrite, PO_4_^3-^: phosphate, SiOH_4_: silicate, POC: particulate organic carbon, PON: particulate organic nitrogen, SPM: suspended particular matter, Chl. a: chlorophyll a, and N/P: nitrate/phosphate ratio.

**Fig 2:**
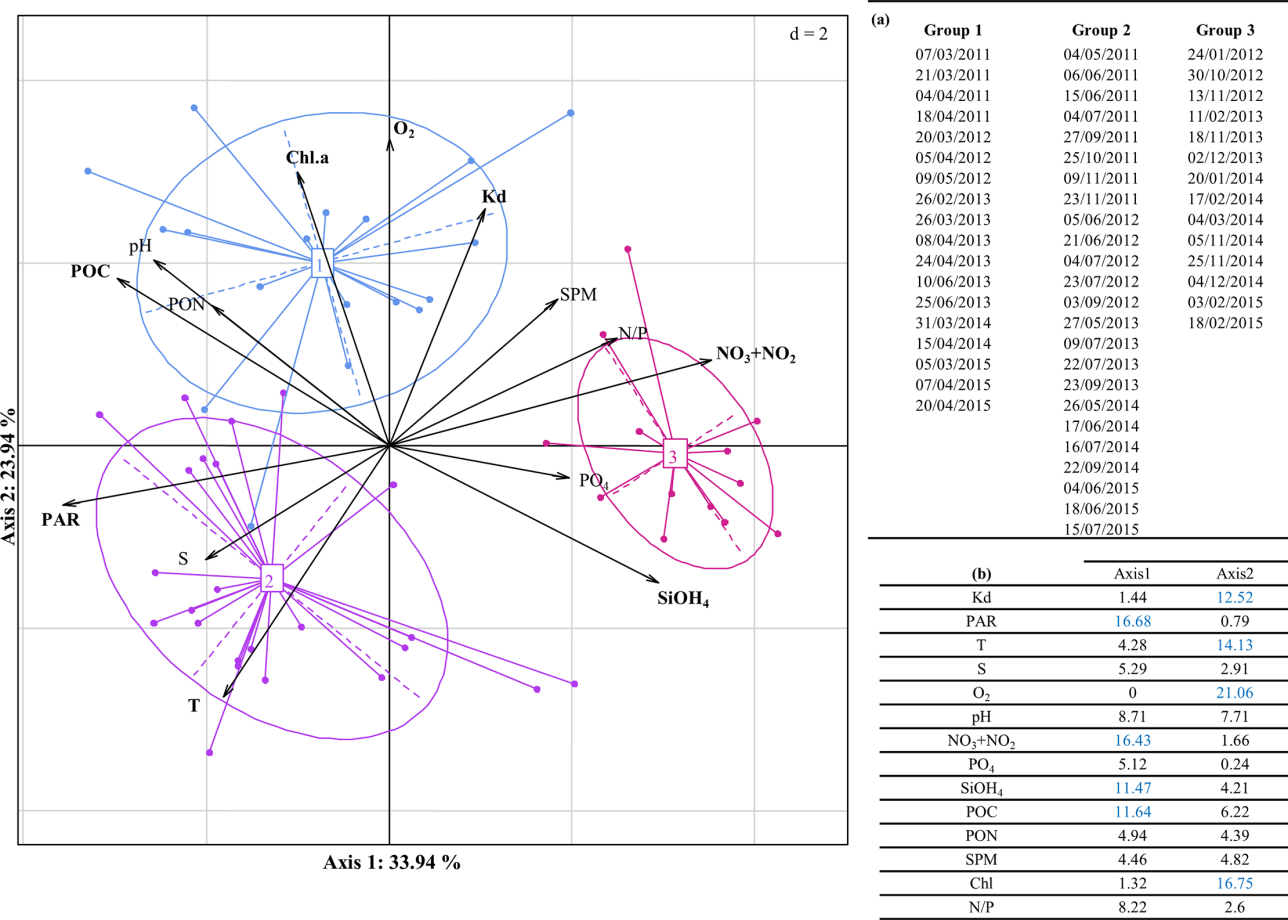
Principal Component Analysis (PCA) applied to the environmental variables recorded at SOMLIT station. Projection of the environmental variables (arrows) and the sampling dates (colored points) on the first factorial plane explaining 58% of the total data inertia. The blue, red and purple ellipses (numbered 1, 2, 3, respectively) correspond to the three groups of sampling dates revealed by the HCA with Ward' method of the PCA dates coordinates on the factorial plane. Most contributing environmental variables to the variability on the first two axes are marked in bold. **(a)** Sampling dates grouping on each cluster **(b)** Contributions of each environmental variable on the first two axes of the PCA in percentage. In blue: strong contributions of environmental variables (>10%).

Principal Component Analysis (PCA) applied to environmental variables showed that the first two components (PCA1 and PCA2) represented 58% of the total variability in the data. All environmental variables except for salinity, pH and SPM, contributed >10% to the formation of the two first axis of the PCA (Fig 2A and 2B). PCA1 strongly correlated to, by decreasing order, PAR, NO_3_^-^+NO_2_^-^, SiOH_4_ and POC, while PCA2 significantly correlated to O_2_, Chl. a, SST, and Kd. The environmental variables PON and PO_4_^3-^ (14% and 32% of total contribution, respectively) contributed mainly to the third PCA (not shown). The Hierarchical Cluster Analysis (HCA) with Ward's method, allowed to distinguish three seasonal periods (Fig 2). The first period corresponded to *P*. *globosa* spring blooms (from March to June), and was associated to high values of Kd, O_2_, Chl. a, and POC, that are characteristics of spring conditions. The second period (from early May to early November) was associated to warm and nutrient-limited waters. Finally, the third period (from mid-November to early March) was associated to low SST and high nutrient levels, typical of winter conditions (Fig 2A and 2B).

### Eukaryotic diversity (rDNA-based)

**Fig 3:**
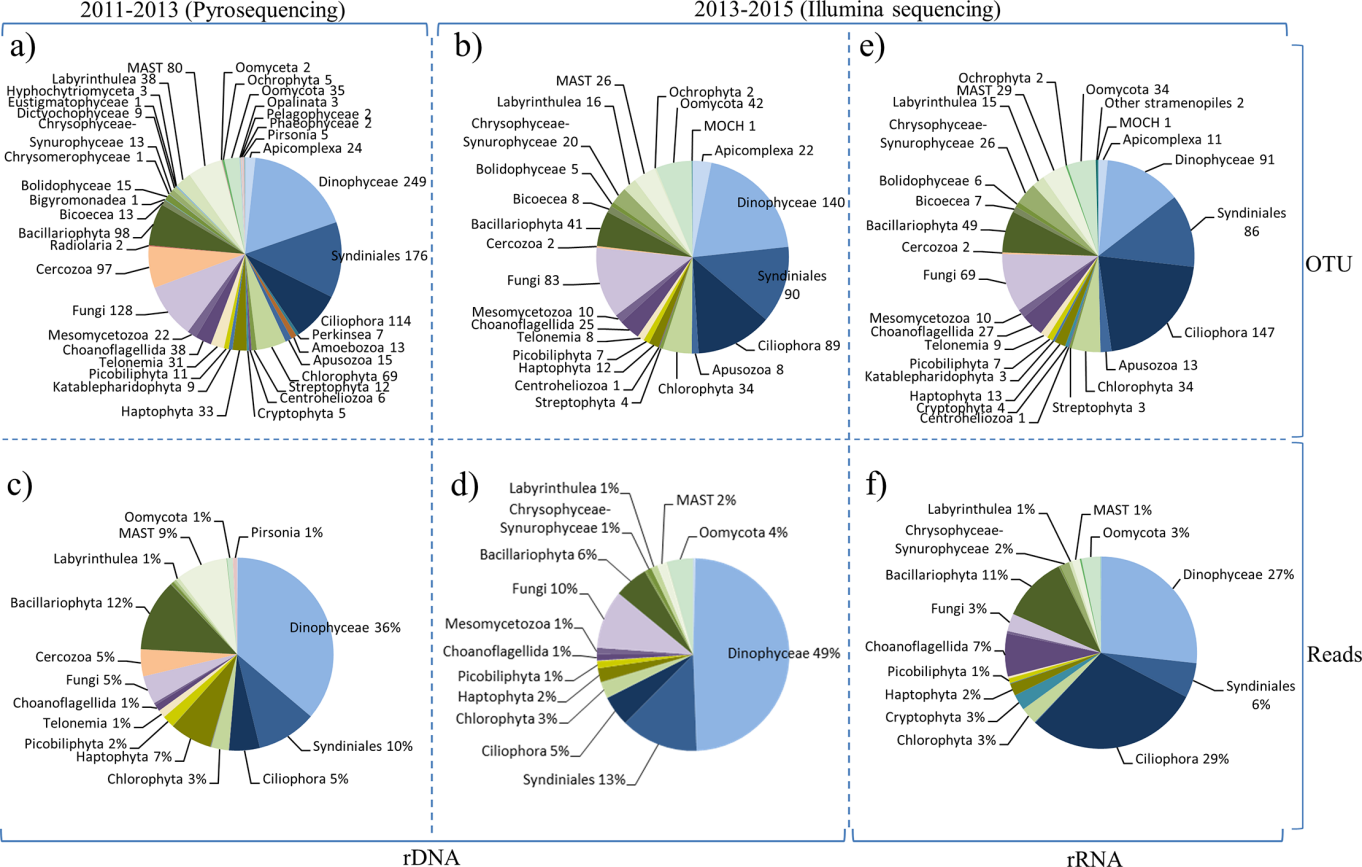
Overview of microbial community structure in the EEC, relative to the studied periods and sequencing methods. Number of OTUs unveiled by pyrosequencing (a) and Illumina (b) methods, and relative number of reads obtained by pyro-sequencing (c) and Illumina (d) methods, based on rDNA sequencing. Number of OTUs (e) and relative number of reads (f) based on rRNA Illumina sequencing. To facilitate reading, the percentage of reads number assigned to a specific group is given when ≥ 1%. Taxonomic affiliation was based on BLASTN searches against the PR2 and Silva databases.

**Fig 4:**
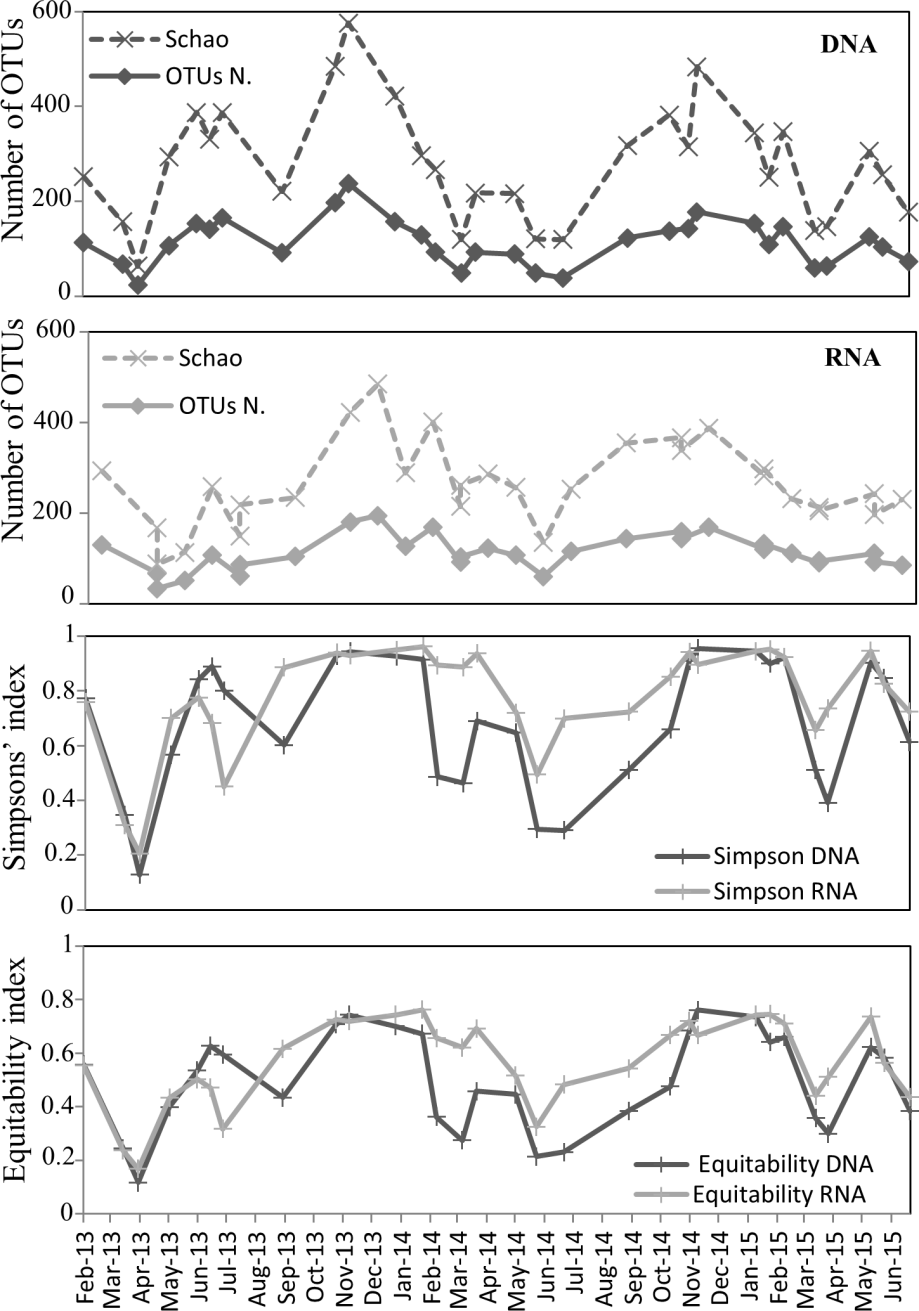
Diversity indices, based on rDNA and rRNA Illumina sequencing, for samples taken in the EEC (2013–2015). Curves represent the number of OTUs (solid lines), the richness estimator (S_chao_1, dash lines) and indices for diversity heterogeneity (Simpson, Equitability). The rDNA-based dataset is depicted with dark grey lines and the rRNA-based dataset with light grey lines.

The DNA-based diversity was conducted during a four-year survey (from 2011 to 2015). During this period, the 454-pyrosequencing method (used during the first survey from 2011 to 2013) was gradually discontinued at the benefit of Illumina sequencing (used during the second survey from 2013 to 2015) due to fast and continuous development of high throughput sequencing technologies. The rDNA-based analysis identified 1388 OTUs with the pyrosequencing approach (47 samples, 2011–2013) and 696 OTUs with Illumina sequencing (30 samples, 2013–2015). Overall, the OTUs identified were affiliated to eight super-groups (Alveolata, Amoebozoa Apusozoa, Archeaplastida, Hacrobia, Opisthokonta, Rhizaria and Stramenopiles) with Amoebozoa only identified during the 2011–2013 sampling period (13 OTUs) (Fig 3A, Table 2). The two datasets were analyzed separately. For both periods and sequencing methods, no significant difference was observed on the OTU abundance and taxonomic distribution of dominant higher taxonomic groups (representing at least 1% of the total OTUs) based on the Kolmogorov-Smirnov test (*p-value* = 0.22). However, a significant difference (Kolmogorov-Smirnov *p-value* < 0.005) was observed between the two sequencing methods and sampling periods when including rare groups. For example, the Radiolaria were only observed in the pyrosequencing dataset (Fig 3A and 3B, Table 2). Overall, among the 40 groups identified (Table 2), 24 were found in both surveys, 15 were found only in the pyrosequencing dataset, and one group (MOCH) was identified only in the Illumina dataset (in May, September, November 2013 and in February 2014) (Fig 3B, Table 2). The highest number of OTUs was consistently found in November-December (with 373 OTUs for 2011–2013 and 238 OTUs for 2013–2015), while the lowest diversity was repeatedly found in April (with 46 OTUs in April 2012 and 24 in April 2013, for the pyrosequence and Illumina datasets, respectively) (Fig 4). Alveolata was always the most diverse group, comprising 570 OTUs (in 2011–2013) and 341 OTUs (in 2013–2015). This group was mostly composed of Dinophyceae (~20% of all the OTUs), followed by Syndiniales (~13%) and Ciliophora (~11%). Stramenopiles (327 OTUs in 2011–2013 and 161 OTUs in 2013–2015) was also a highly diverse group composed of Bacillariophyta (~6% of all the OTUs), MAST (~4%) and Oomycota (~6%). Finally, Fungi represented around 10% of all OTUs (Fig 3A and 3B). Although the number of reads can be only considered in terms of relative and not absolute abundance, it can be noted that Dinophyceae showed the highest reads number (36% and 49% of the total number of reads, in the 2011–2013 and 2013–2015 surveys, respectively), followed by Syndiniales (10% and 13%), Bacillariophyta (12% and 6%), Fungi (5% and 10%) and Ciliophora (5% and 5%) (Fig 3C and 3D). Overall, significant differences were found in the distribution of taxonomic groups read’s abundances (Kolmogorov-Smirnov *p-value* < 0.001) between the two surveys. For example, pyrosequencing generated a higher proportion of reads for MAST (9%) and Haptophyta (7%) compared to Illumina (Fig 3C and 3D).

**Table 2:** Taxonomic groups unveiled in the EEC from 77 samples either by pyrosequencing (2011–2013, 47 samples) or by Illumina sequencing (2013–2015, 30 samples).

	2011–2013	2013–2015
Groups	Pyrosequencing	Illumina
**Alveolata**	**✓**	**✓**
Apicomplexa	✓	✓
Dinophyceae	✓	✓
Syndiniales (MALV)	✓	✓
Ciliophora	✓	✓
Perkinsea	✓	✓
**Amoebozoa**	**✓**	ND
**Apusozoa**	**✓**	**✓**
**Archaeplastida**	**✓**	**✓**
Chlorophyta	✓	✓
Streptophyta	✓	✓
**Hacrobia**	**✓**	**✓**
Centrohelioza	✓	✓
Cryptophyta	✓	ND
Haptophyta	✓	✓
Katablepharidophyta	✓	ND
Picobiliphyta	✓	✓
Telonemia	✓	✓
**Opisthokonta**	**✓**	**✓**
Choanoflagellida	✓	✓
Mesomycetozoa	✓	✓
Fungi	✓	✓
**Rhizaria**	**✓**	**✓**
Cercozoa	✓	✓
Radiolaria	✓	ND
**Stramenopiles**	**✓**	**✓**
Bacillariophyta	✓	✓
Bicoecea	✓	✓
Bigyromonadea	✓	ND
Bolidophyceae-and-relatives	✓	✓
Chrysomerophyceae	✓	ND
Chrysophyceae-Synurophyceae	✓	✓
Dictyochophyceae	✓	ND
Eustigmatophyceae	✓	ND
Hyphochytriomyceta	✓	ND
Labyrinthulea	✓	✓
MAST	✓	✓
*MOCH*	ND	✓
Oomyceta	✓	ND
Ochrophyta	✓	✓
Oomycota	✓	✓
Opalinata	✓	ND
Pelagophyceae	✓	ND
Phaeophyceae	✓	ND
Pirsonia	✓	ND
Other Stramenopiles	✓	ND

Groups identified only between 2011 and 2013 (pyro-samples) are underlined. The MOCH group (in italic) was only identified between 2013 and 2015 by Illumina sequencing. ND: Not Detected.

### Microbial community composition and seasonal succession revealed by rDNA and rRNA sequencing

Between 2013 and 2015, a total of 696 OTUs and 700 OTUs were detected by rDNA- and rRNA-based Illumina sequencing, respectively (Fig 3B and 3E). The rDNA- and rRNA-based diversity followed a similar pattern of OTU abundance (Fig 4). The mean ratio of observed to expected OTUs (Chao-1) was 69±8% for DNA samples and 78±7% for RNA samples. The Chao-1, Simpson and Equitability indices reflected the same pattern, with relatively higher values from October to February, and lower values during *P*. *globosa* bloom (from mid-February to May) (Fig 4). Over 60% of total OTUs were shared between the rDNA and rRNA datasets, whereas 168 OTUs were only identified in the rDNA dataset and 172 OTUs were only identified in the rRNA dataset. A total of 868 OTUs corresponding to 26 higher taxonomic groups were found when considering both rDNA and rRNA datasets (Fig 3B and 3E). The proportion of OTUs affiliated to these major super groups was similar whether considering the rDNA or rRNA dataset (~49% of Alveolata, ~23% of Stramenopiles, ~15% of Opistokonta, 4~5% of Archaeplastida and Hacrobia, and 1~2% of Apusozoa) (Fig 3B and 3E). In the rRNA dataset, the most diverse group was the Ciliophora (147 OTUs gathering together 29% of the total reads), with the majority being affiliated to the Spirotrichea (60 OTUs) (Fig 3E and 3F). The second most diverse group was the Dinophyceae (91 OTUs, 27% of all reads) followed by Syndiniales (86 OTUs, 6% of all reads), Fungi (69 OTUs, 3% of all reads) and Bacillariophyta (49 OTUs, 11% of all reads) (Fig 3E and 3F). No significant difference was observed between the rDNA and rRNA datasets regarding OTU abundance and taxonomic distribution for higher taxonomic groups (Kolmogorov-Smirnov p-value = 0.98).

**Fig 5:**
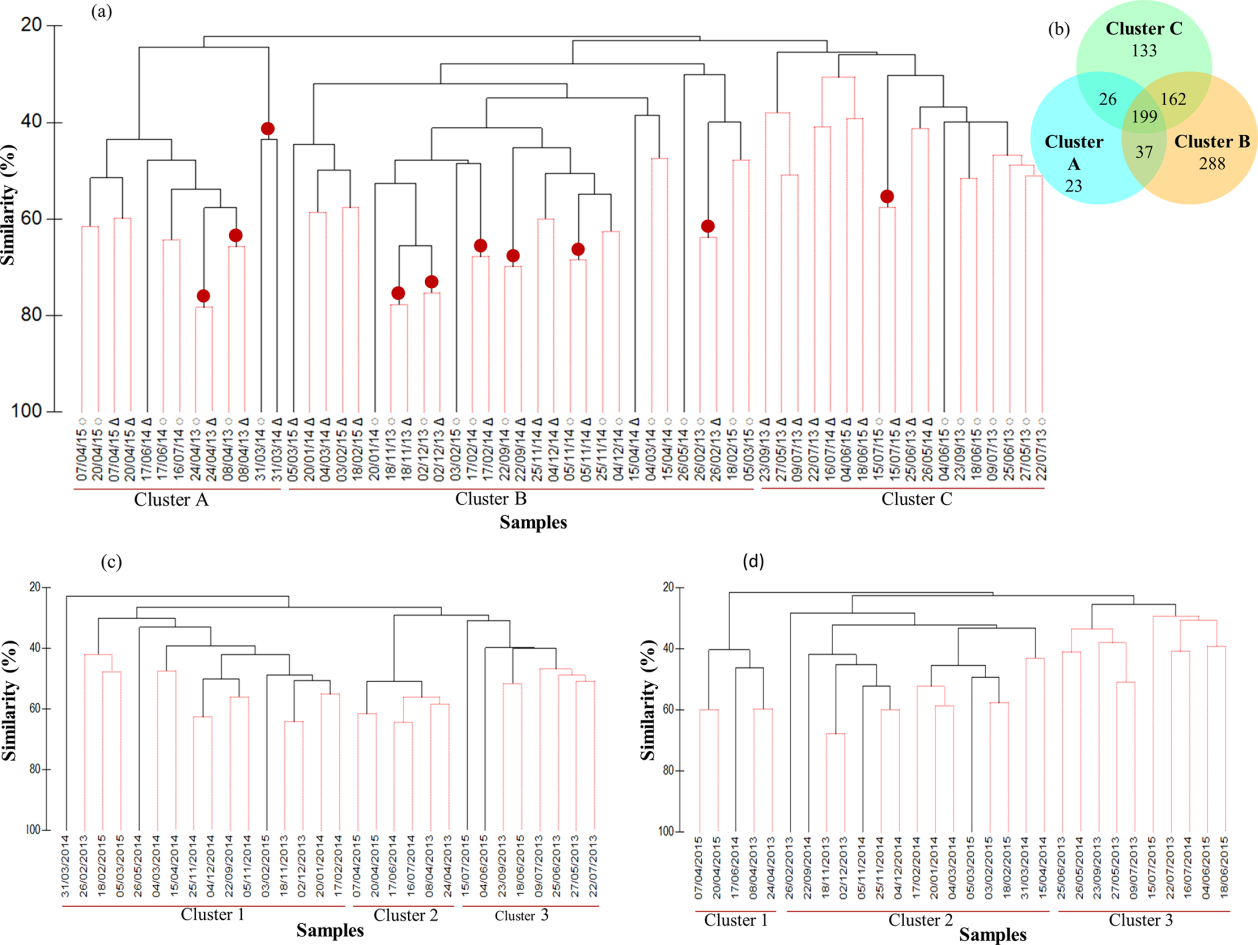
Cluster-based microbial community structure in the EEC. (a) Cluster diagram based on Bray–Curtis dissimilarities calculated based on the non-transformed number of reads for all OTUs found during the study. Red lines in the dendrogram indicate significant differences (p > 0.05) between bifurcations, based on the SIMPROF significance test. ◌ DNA samples, Δ RNA samples. Red dots indicate grouping of samples from the same sampling date (b) Venn diagram of shared diversity between three clusters. The total richness for all groups was 868 OTUs. The number of species shared between the three clusters was 199, corresponding to 23% of the total richness. Similar Bray-Curtis cluster diagrams were constructed from rDNA-based (c) and rRNA-based (d) number of reads.

The cluster analysis applied together on the total read abundance of OTUs from rDNA and rRNA samples showed three main clusters at a similarity level of 25% (Fig 5B). The first cluster (Cluster A) grouped mainly samples from March to July (including the bloom period of *P*. *globosa*). The second cluster (cluster B) grouped autumn and winter samples (September to March). Finally, the third cluster (cluster C) included summer and early autumn samples (late May to early September). Protist community structure based on rDNA and rRNA sequencing exhibited minor differences. In total, 10 of the 30 DNA/RNA pairs (i.e. rDNA and rRNA from the same date) appeared side by side in the dendrogram (red dots in Fig 5A), and 17 of the 30 pairs belonged to the same cluster at 40% similarity. The remaining pairs of samples showed lower similarity, but they still belonged to the same seasonal cluster, except for two samples (July 16^th^, 2014 and May 26^th^, 2014) (Fig 5A). The Venn diagram showed that only 23% of OTUs were shared among the three clusters (Fig 5B). While fall-winter (cluster B) and summer (cluster C) periods had relatively distinct diversity (~43% of shared OTUs), the blooming period of *P*. *globosa* (cluster A) shared most of its diversity (>90% of OTUs) with the other two clusters (Fig 5B). When the same cluster analysis was applied to total OTU read abundance from rDNA (Fig 5C) and rRNA (Fig 5D) separately, the clusters inferred from rRNA data showed a clearer seasonal representation of the community succession than the one based on rDNA. In fact, 79% of the rRNA samples (compared to 72% for the rDNA samples) belonged to a cluster in agreement with those that were identified in the PCA of environmental variables (Figs 2 and 5C and 5D).

### Temporal variation of abundance and activity of most abundant OTUs

**Fig 6:**
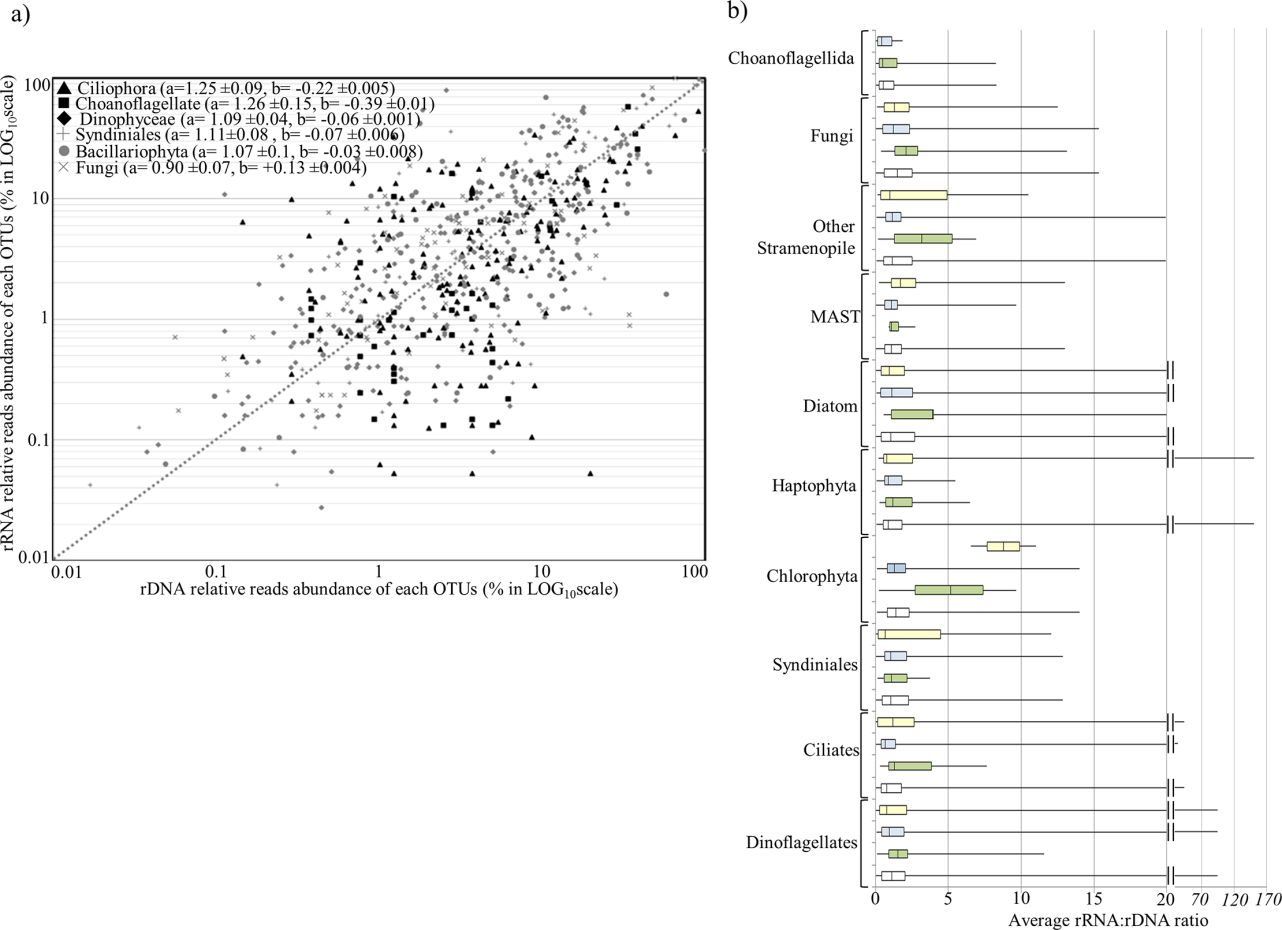
Relation between abundance (rDNA reads) and relative cell activity (rRNA reads) for the 100 most abundant OTUs. (a) Each OTUs was plotted according to their relative number of rDNA (x-axis) and rRNA (y-axis) reads. Each point represents paired percentages (in logarithmic scales) of each taxonomic group from each sample. (b) Boxplot representing rRNA:rDNA ratios for the major taxonomic groups of the 100 most abundant OTUs. The boxplots represent the variation of the rRNA:rDNA ratio around the median for each seasonal clusters (white: all years, green: cluster A representative of *P*. *globosa* bloom period, blue: cluster B corresponding to winter conditions, yellow: cluster C corresponding to spring-summer conditions). Whiskers indicate the maximum and minimum values.

The rRNA:rDNA ratio was used as a proxy of relative cellular activity for the 100 most abundant OTUs (>0.1% of the total reads abundance) which represented over 90% of all reads. Despite of important variability, the relative abundance of each OTU (rDNA-based dataset) generally increased with cellular relative activity (rRNA-based dataset) (Fig 6A).

#### Dinoflagellates (mostly heterotrophic micrograzers and mixotrophs)

This group represented almost half (48%) of the 100 most abundant OTUs’ sequences. The large number of rDNA reads for this group is related to high rDNA gene copy number present in the genome of Dinoflagellates [65,66]. The most abundant OTU during the entire survey was affiliated to *Gyrodinium spirale* (OTU1: 35% of all sequences) and was found in all samples. Three OTUs (OTU1, OTU2 and OTU10), having *G*. *spirale* as their closest relative, all reached recurrent highest abundance in April-June (Table 3). *G*. *fusiforme* (OTU21) followed the same seasonal succession as *G*. *spirale*. The second most abundant dinoflagellate was *Torodinium robustum* (OTU61), mostly present from September to February (Table 3). The rRNA:rDNA ratio (as a proxy of cells activity) for dinoflagellates was generally low, but showed some increase in spring compared to the rest of the year (Fig 6B). In general, the number of rRNA reads appeared to be lower than rDNA reads, suggesting that dinoflagellates were abundant in the EEC but with a relatively low cell activity (Fig 6B). *Warnowia sp*. (OTU4) and *Katodinium glaucum* (OTU8) were present all year long. *Warnowia sp*. displayed its highest abundance and higher rRNA:rDNA ratio from September to February, while peak abundance of *Katodinium glaucum* was detected in June/July. Other unclassified Dinophyceae (8 OTUs) were detected throughout the year with seasonal preferences depending on the OTU. The most abundant unclassified Dinophyceae sp. (OTU3) was observed from March to July with a peak of activity in March or June. Another Dinophyceae sp. (OTU7) was found all year with a peak of activity in September, and an increased abundance in November. Several other Dinophyceae sp. (OTU24, 126, 131 and 245) were also mostly detected between November and February (Table 3).

**Table 3:** Heat-map showing abundance (rDNA-based survey) and relative activity (rRNA/rDNA ratio) of the 100 most abundant OTUs (representing more than 92% of all the reads) detected between 2013 and 2015. Positive *versus* negative ratios can be used as an indication of relative cell activity. Negative rRNA:rDNA ratio indicates low cell activity, in contrast to positive ratio which corresponds to higher cells activity (for example, a ratio of -40 corresponds to an OTU having 40-time more rDNA reads than rRNA reads).

		2013										2014												2015							
OTU	Affiliation	26 feb.	8 Apr.	24 Apr.	27 May	25 Jun.	9 Jul.	22 Jul.	23 Sep.	18 Nov.	2 Dec.	20 Jan.	17 Feb.	4 Mar.	31 Mar.	15 Apr.	26 May	17 Jun.	16 Jul.	22 Sep.	5 Nov.	25 Nov.	4 Dec.	3 Feb.	18 Feb.	5 Mar.	7 Apr.	20 Apr.	4 Jun.	18 Jun.	15 Jul.
**Dinoflagellates**																															
1	*Gyrodinium spirale*	1.4	1.9	1.7	-33	1.4	-8.9	-2.5	10.8	1.4	2.0	-9.4	-2.8	-3.5	1.2	-4.3	2.4	1.5	-3.1	1.3	1.2	-3.3	-2.4	-3.6	-16	-3.0	1.5	1.2	-4.2	-27	2.0
2	*Gyrodinium spirale*		1.9	2.2	-	2.3	-	-3.1		3.4	1.7	-		-		1.1		2.0	-	1.8	1.7	-	-3.6	-		-	-2.1	1.2	-1.4	-	
3	Dinophyceae sp.	-			-	1.1	-	-1.3	-								10.5	2.2	-		-			-	-	-	-	1.8	-2.8	-4.6	3.4
4	*Warnowia* sp.	-1.1	-2.1	-	-	-	-3.6	-1.5	6.2	1.9	3.0	-8.0	1.2	-1.1		-4.6	3.3	-1.8	1.4	2.4	2.5	-1.6	-2.3	-1.8	-2.9	1.9		1.4	-	-	7.0
7	Dinophyceae sp.	1.8	1.8		1.4	-	-	-2.2	3.8	1.3	1.9	-14	-1.1	-5.9	11	-2.0	-2.7	1.4		2.6	1.3	-1.8	-2.3	-1.3		-	1.4	2.4	-	-1.8	2.0
8	*Katodinium glaucum*	2.3	2.1	2.1	-63	1.2	-10	-6.2	-8.0	2.0	1.7	-		-	-	-	94	-1.0	-2.3	-1.1	1.4	-3.8	1.1					-	-6.2	-	2.7
10	*Gyrodinium spirale*		1.7	3.3	-	-2.9	-	-			-							1.8	-6.6								-1.2	-1.1	-2.2		
21	*Gyrodinium fusiforme*		-1.6	3.1	-	-1.1	-	-1.5			-					-		-1.1	-							-	-3.3	-1.8	-		
24	Dinophyceae sp.	-1.6	3.7			-	-		1.2	1.5	2.4	-5.3	2.6	-5.7		-4.9	-6.5			2.9	2.0	-2.0	-2.5	-1.6	-2.5	-	2.0	1.4	-	-	
32	*Pelagodinium beii*	1.7	3.8			-		-	-			-	1.3			-	-4.0	-2.0	-			-	-	1.3	-				-	-	6.3
51	*Gyrodinium* sp.	-2.2	-10	-	-	-	-	-5.4	30	-2.5	-1.3	-	-1.8	-		-3.1	1.2	-		2.7	-2.1	-1.8	-1.8	-14	-				-	-	-1.8
60	*Heterocapsa pygmea*	3.0	-		-		-			1.8	5.6	-4.2	3.5	1.1		1.4	-		-	3.1	-1.6	2.8		-1.4	-		2.8				1.1
61	*Torodinium robustum*					-1.1	-	-	3.8	-1.2	2.9	-	-2.7	-1.1		-				2.8	2.5	-11	-2.9	-						-	
70	Dinophyceae sp.		6.2		-	1.3	-	-2.3		-							28.7			-										-	
76	*Gyrodinium* sp.	-			-1.5		-		39	1.1	-1.4	-12	-1.2	-1.7	2.6	1.1	-1.3	4.6			-		1.3	-		-	4.9	1.2			
113	*Chrysophyceae* sp.	-						-		-1.0	-1.6		-1.0							1.7			-11	-		20					
121	*Azadinium cuneatum*												1.1	1.4		-							-								
126	Dinophyceae sp.		-		-		-	1.3	-3.8	2.0	2.4	-2.4	1.4	-3.4	-	-	4.9	5.8		5.8	2.4	-1.1	-2.2	2.0							-1.0
131	Dinophyceae sp.		-		-	-		-				-					-			4.6	2.8	-3.9	-2.2	-						-	
167	*Gephyrocapsa oceanica*	1.0	-		-	-	-	-1.4	-	3.6	2.0	-3.1	-	-1.2				-	1.0	-	4.0	-	4.0								
209	Chrysophyceae sp.	1.2			-	-	-			1.5		-		1.8	2.9						1.2	2.3	-2.4			4.3		-3.4			
245	Dinophyceae sp.											-		6.6	-						1.4	-1.4	-1.8	-							
256	Dinophyceae sp.																-4.0														
**Ciliates**																															
5	*Strombidium basimorphum*	1.3	-1.4	-	1.3	1.9	-38	1.2	-7.9	-1.1	-1.8	-1.7	-2.4	1.7	7.6	3.0	-7.0	-		1.3	-1.6	-1.8	-1.3	-2.3	33	3.6	-1.1	3.3	43	7.4	
12	*Lynnella semiglobulosa*	-	-		-23	-82	-71	-1.0	-378	-9.5	-28	-3.0						5.7		-7.9	-2.0	-2.3	-1.8							3.3	
44	*Strombidiidae* sp.	1.4	-3.0	-		-		-	1.4	-1.5	1.7	1.3	-2.1	4.0		2.4	-			-	-2.8	-2.0	1.5	-5.4	4.9	-2.5		1.3			
56	*Cyclotrichia* sp.	-			-			-1.3		-14	-7.7	-1.5	-6.1	-1.6	-1.1		-16					1.6	-16	-1.4	11	6.3	3.8	6.5	2.4		-4.1
64	*Pelagostrobilidium neptuni*	-			2.7		26		-3.2	-5.8		1.0	-4.1	-1.7		-1.5	-					-7.0			-1.3		-		2.6	3.6	-2.3
78	Choreotrichia-1 sp.	-	-		-	-				-1.5	-1.3	1.1	-1.6	1.2	1.1		-				1.5		-2.2	1.1	10	10			-		
84	*Parastrombidinopsis shimi*										1.1		8.5	3.4										6.8	-1.7						
91	*Salpingella* sp.				-	-	-	-16	-32	-9.4	-3.0	-1.1	-1.8				-			-2.1	-2.7	-8.0	-2.7	1.1	14					3.7	
92	*Strombidinopsis acuminata*						-	-16		-4.4	-3.1	1.4	1.3								-2.0	1.5	2.0			-7.5					
112	*Strombidiidae* sp.	3.0	1.3		4.6	-	-2.8			-1.4	-1.6	-1.3	-1.2	-1.4		4.2					-2.8	-1.3	-1.5	-6.6	1.4	-	-1.0	1.8	13		
141	*Pelagostrobilidium* sp.					2.4	1.3			-3.3	-1.1	-									4.2	-1.9	-2.2	-							
166	*Pseudocohnilembus persalinus*										4.8		19	-					-	7.2		-	-2.0	1.1	-	-		1.2		1.2	
409	*Laboea strobila*				4.8	-			-1.4	-15	-11	-3.3	-1.6	-1.0	4.1	-1.7					-2.4	-1.4	-		-1.3			-1.1	2.0	1.7	
**MAST, Choanoflagellates**																															
6	*Stephanoeca cauliculata*	-8.8	-				-3.2		-11	-28	-19	-3.8	-3.7	-1.1		8.3	-4.1			-38	-3.6	1.5	-1.5	-1.9							
18	MAST-7C sp.	1.0			-	-2.0	-	-4.0	13	1.8	1.1	-	1.7	1.0	1.0	-1.7	5.0			1.4	-	-2.7	-3.0	-1.6	-	-1.4	1.2		-	2.0	-
52	MAST-1A sp.	2.3	-							-			1.1			-1.5						-				-29					
94	*Calliacantha natans*				-	-6.4	-1.6	-	-25	-2.4	-3.8	-3.8	-2.3	-1.7		5.7				-2.1	-2.6	-1.4	-1.1	2.9							
96	MAST-1B sp.	-			-	-				-1.5	1.2	-	-		2.7	1.3	-					-		-5.5			-1.1			1.8	
130	*Stephanoecidae* sp.	3.2				1.9	-	-		-3.2	-2.1		-2.8			1.7	1.5	-		3.8	-1.1	2.5	-1.2	-3.3	3.8	-1.6		-	1.3	-1.2	
201	MAST-2C sp.				-	1.6	-				9.7					1.1	1.3			-	9.7		-					-			
**Phytoplankton**																															
13	*Chrysolepidomonas* sp.	1.3					-3.8	-3.8		-	1.3		1.3	-	3.9	-	10			3.3	1.3	-6.1	-1.1	-2.3	-3.1				3.9		
14	*Chrysochromulina leadbeateri*	-2.8			-4.3	-		-3.1	-1.6	1.8	-1.0	-1.1	-1.1	3.2	6.5	-1.2	2.0			1.5	1.5	-1.4	-1.4	-1.7	3.8	-2.0	1.6	-	-1.9	-1.2	
16	*Leptocylindrus aporus*	-2.4		1.3	8.2	1.0	2.0	-1.6	-36								29.0											-1.8	2.3	1.6	-
17	*Thalassiosira hispida*							-		-4.1	-11	-4.4	-4.1	2.3							-6.2	-12		-4.9	6.5	2.3					
**27**	***Phaeocystis globosa***	-2.9	-2.3	-3.5	-1.4	-		-	150	-1.3	1.3	-10	1.2	-2.3	5.2	-1.9	-			1.6	2.0	-4.2	-10	-6.4	-1.2	-1.2	1.4	-1.3	3.5	-	-
36	*Haptolina fragaria*		-		-	-	-	-	-4.0	3.6	1.8	-1.5	-	-			5.5			-	1.8	-1.5	-						2.7	-	
41	Chlorellales sp.	-10	-			-				1.8	-1.2	7.3		7.3					11	-	-	5.5	11	2.0	-1.2	-1.3	-				
47	*Ditylum brightwellii*							-		2.2	2.6	-3.0	3.3	1.9	1.1						3.3	-		3.7	2.3	-1.7					
54	*Bathycoccus prasinos*	-1.2	-		-	-		-	-	-1.0	1.3	1.1	-1.4	1.9		4.8	-			-1.2	1.9	-1.2	-1.2	-1.2	14	12				-	
66	*Micromonas pusilla*	-			-	-		-	-	1.6	-1.9	1.7	1.1	5.5		1.1	-			1.1	1.4	-1.3	-1.8	-1.5	5.8					-	
86	*Polar-centric-Mediophyceae* sp.	3.9			-	-		-		2.5	1.3		-2.0								4.3	-2.0	-2.7						-	-	
88	*Guinardia delicatula*							-1.0	-3.6	2.0	2.8			-		-	-	4.0		-	8.0	-1.5	1.7			-	4.0	4.0	-	-1.9	
103	*Picomonas judraskeda*		-			-				2.0	-1.0	-	1.5	-		-1.8				3.3	1.1	-3.7	-4.3	-1.8	-	3.3	-		-		
104	Picobiliphyta sp.	-2.6	-1.3		-	-	-	-2.1	2.8	-1.1	1.8	-7.8	-2.1	-		-2.4	50			2.1		1.0	-2.3	-	-	-		3.6	-1.3	-	
110	*Pycnococcus provasolii*	1.4	1.6			-		-	-	1.6	2.2	-	2.5	-	-	2.5	-		6.5	-1.1	1.6	-	6.5	1.6	-4.0	-		-	-	-	
132	*Guinardia flaccida*				3.0	-3.7	1.1	-5.5	-1.7							-								-		1.3	-	1.0	-2.8	-7.4	-3.7
168	*Bolidophyceae* sp.					-				-1.2	-3.3	-1.2	-1.3								-1.8	1.7	-	4.3	1.1	1.5	1.4				
213	*Micromonas pusilla*					-			-	1.2	-1.7	2.5	1.7	1.4						-2.8	-3.0	-1.8	1.3	1.3							
221	*Eucampia* sp.				-			1.0	-	-7.3	-14				6.5						-1.6	-1.8	-4.9						3.1	-1.7	
266	*Guinardia delicatula*	-1.8			-1.8	-	-	-2.6	6.7						3.9	-1.1												-			
370	Picomonas sp.	-				-		-		2.9	-2.5									1.8	3.7	1.2	-5.3								
581	*Guinardia flaccida*	-					-	1.9	-			-			23	11		-						-				-1.4	-	-1.1	
1085	Picobiliphyta sp.				-	-	-	-2.4	-	4.4	1.5						22.0	-		4.4										-	
**MALV, Fungi and other potential parasites**																															
9	*Olpidiopsis porphyrae*						-	-	-	-1.0	3.4	-			2.7	1.3				-1.1	-1.1	-3.9	-5.2	-							1.0
11	Dino-Group-I-Clade-1 sp.	2.6	1.2	2.5	-5.7	-3.2	5.2	-	6.7	2.3	2.8	-23	-1.2	-2.7	3.7	-1.0	-18	1.0	-6.3	2.1	1.8	-1.4	-1.4	-3.0	-20.3	-6.0	1.1	-2.2	-8.4	-	-2.2
15	*Haliphthoros* sp.	3.7	-5.2	6.9	-1.7	-1.7	-	-		2.8	2.3	1.1	-1.5	-		-			6.9				-	-7.6	-14	-	3.4	-	1.3	-	-
19	*Xenomeris raetica*	4.8	-		-	2.4	-	-	-	1.2	4.8	9.6				-	-			-			2.4	-1.7	-	-7.5	-1.5		-	2.4	3.2
20	*Penicillium* sp.	-	2.9	-	-2.9		-		-		-	-		-	-1.0			-	2.3	-		-	2.9	-	-	-2.4	1.2		-	-	
23	*Ichthyosporea* sp.	-	-		-	1.3	-	-2.2	-	1.9	3.7	1.2	7.4	-	6.2	-	-10			1.9	-	-	-	-	3.7	-	3.4		-	-1.7	3.2
31	*Cryptomycotina* sp.	1.5								-	-		-											-	-	-	-		-		
35	*Engyodontium album*	1.8	2.7	4.2	-2.2	1.1	-	-1.6	-	2.8	-1.0	-1.9	-1.9	-2.6	2.9	-	9.7	4.2	12.5	4.2		1.3	-1.1	-2.1	-8.6	-3.7	2.5	5.0	-2.0	-	-1.2
42	*Xenobotrytis acaducospora*	2.1	1.6		-					-		-			1.7	-			-2.7			-	-	-1.9	-	-6.4	-1.3	1.4	-10	-	-1.3
45	*Thraustochytriaceae* sp.				-1.0	5.0	-5.4	-2.4	-1.9		-						9.7	-1.0	4.9					-	-	-	-		-3.2	-1.7	1.8
48	*Gregarines* sp.						-																		-1.1						
53	*Meira miltonrushii*	15	1.5	13	-	2.2	-	-		1.6	1.9	-32			-1.1			-	1.9		2.2	-1.4	-	-	-	-2.1	2.2	2.7	-1.4	-	1.5
58	Oomycota sp.	1.2										1.1			-							1.3	-	-	8.3	-1.1	-				
62	*Abeoformidae* MAIP 2 sp.		-1.3					-1.1	-	-4.3	2.5	-					-1.1	-	-2.3	-1.1	7.5	2.4	1.1	-3.7		-				-1.2	1.2
72	*Thraustochytriaceae* sp.				-	7.4	-							1.9		-	-					-				-2.4			-6.5		
74	*Achlya bisexualis*	-2.3																								1.1					
87	*Gregarines* sp.				-	-				-	3.7		-					-											-		5.8
98	*Pseudoperkinsus tapetis*			3.0	-	-	-	-2.0								-		5.9		-				-	-	1.5	3.5	8.9	-	1.5	-
150	*Abeoformidae* MAIP 2 sp.													-1.4	-	1.1			-1.8		-								-		
155	Dino-Group-II-Clade-13										-				-		-			1.6	1.6	1.5	-1.5	-			-	-3.6	-		
173	Dino-Group-II-Clade-22									7.0	7.0	-		-								-1.1	-1.3	-							
181	Dino-Group-III sp. strain1					-			-	-1.5	1.5		1.2				-15			-	5.4	-	-8.8	-					-	3.7	
184	*Olpidiopsis* sp.	-1.4																							2.7	1.2					
186	Dino-Group-I-Clade-1 sp.	1.1	-		-	-		-		1.4	1.1	-																			
215	Dino-Group-I-Clade-1 sp.				-	-	-	1.6		5.1	2.6							3.4		-	13								-		
220	Dino-Group-I-Clade-1 sp.	2.7	1.1		-	-	8.6					-5.1	-	-						4.2	1.1	-2.8	-3.9	-2.1	-	-			-	-	-1.1
229	Dino-Group-II-Clade-8 sp.				-	-1.5	-	-		2.7	-1.5			-			12			3.6	1.5	-2.2	-3.6								
265	*Chytridiomycetes* sp.	2.8	-					-			1.7	-												-5.6	-39	-					
281	Dino-Group-II-Clade-10-and-11 sp.									-1.3	-1.3	-								-1.1	1.8	-	-								
294	*Exobasidiomycetes* sp.	-2.0			-	2.0				-1.3	12		-	-		1.7	-	-		-				1.3	-	-	2.0	-	-		-
352	Dino-Group-II-Clade-10-and-11 sp.	-								1.0	1.1			-							2.9	-1.1	-11	-							
361	*Haliphthorales* sp.					-	-	-7.1											6.4												
519	*Cryptomycota* sp.																							-	-	-					
638	Dino-Group-I-Clade-5 sp.					-		-		2.0	-1.0									1.5	2.2	-1.1	-1.8	-							-
Legend		0%			<0% to 1%			<1% to 5%			<5% to 10%			<10% to 25%			<25% to 50%			<50% to 75%			<75%		

OTUs presented here accounted for 92% of total reads and are displayed according to their taxonomic group’s affiliations. Grey shade represents the relative abundance (rDNA-based) of each OTU within each sample, white box indicate absence while darker grey represent higher abundance (see ribbon). Values in each box represent the rRNA:rDNA ratio (for calculation see material and method section).

#### MALV, fungi and other potential parasites and decomposers

This group constituted about a third of the 100 most abundant OTUs (~27% of the sequences). Diversity was highest in winter and lowest during the spring bloom of *P*. *globosa*. No clear seasonal pattern was observed for this group (Table 3). The most abundant OTU of the group was Syndiniale *GroupI-Clade1* (9.9% of all sequences), which appeared mainly from September to February. More generally, Syndiniales from groups 1, 2, and 3 displayed their highest activity in September/November, just before their peak of abundance that occurred in November/December depending on the year (Table 3). A similar pattern was observed for *Thraustochytriaceae*, with increased activity happening prior the highest abundance that occurred between May and September. Overall, throughout the year it was possible to reconstitute the following succession pattern: *Chytridiomycota* thrived in winter conditions; *Olpidiopsis sp*. (OTU184) appeared only in February; *Pseudoperkinsus tapetis* was observed from February or April until September depending on the year; *Thraustochytriaceae sp*. appeared from May to July; *Haliphthorales sp*. was almost exclusively found in July; *Olpidiopsis porphyrae* was abundant from July to February; *Cryptomycotina sp*. was identified during winter conditions from November to March depending on the year (Table 3). *Engyodontium album* and *Ichthyosporea sp*. were found year round in almost every sample during the study period. Overall, their rRNA and rDNA relative reads abundance plotted close to the 1:1 line (Fig 6A), but this linear relation decreased to 0.9:1 for fungi. Except during their winter peak, fungi showed lower potential activity than the rest of this community (Fig 6A and 6B, Table 3).

#### Ciliates

This group represented around 4% of the 100 most abundant OTUs’ sequences. The most abundant organism, observed all year long, was *Strombidium basimorphum* (OTU5). Other Strombidinopsis (OTU44, OTU92 and OTU112), along with *Choreotrichia* sp. (OTU78), *Cyclotrichia* sp. (OTU56), and *Pelagostrobilidium* sp. (OTU141) were also abundant between November and March. In contrast, two OTUs, affiliated to *Parastrombidinopsis shimi* and *Pseudocohnilembus persalinus*, were present during a narrower period, from December to February with high abundance and activity (Table 3). All these ciliates are nano-grazers feeding on small sized phytoplankton. Overall, the most abundant ciliate OTUs displayed high rDNA and low rRNA reads number, possibly corresponding to species with high abundance but relatively low cell activities (Fig 6A). Higher relative activities for ciliates were observed during the spring and summer period (Fig 6B).

#### MAST and choanoflagellates

This group accounted for about 2% of the 100 most abundant OTU sequences and was restricted to Marine Stramenopile (MAST) and Choanoflagellida. The Choanoflagellida, including its most abundant OTU, *Stephanoeca cauliculata* (OTU6), were mostly observed between September and February, with increasing rRNA reads in the middle of this period (Table 3). The most abundant MAST-7C (OTU18) appeared in nearly all samples, with the highest cell activity in September and the highest abundance in December. MAST-1A (OTU52) peak of abundance occurred in February/March, MAST-2C was mostly present from April to early July, while MAST-1B showed irregular abundance peaks from May to June and from November to February (Table 3). Overall, higher MAST cells activity was restricted to the summer period (Fig 6B).

#### Phytoplankton (diatoms, nano and picophototrophs)

This group accounted for 9% of the 100 most abundant OTUs. The most abundant OTU of this group was affiliated with the diatom species *Leptocylindrus aporus* (OTU16: 3.4% of all reads) and was detected between April and September. The increase of *L*. *aporus* rDNA and rRNA reads displayed similar temporal pattern, but with earlier increase of rRNA reads (Table 3). *L*. *aporus* (OTU16), *Guinardia flaccida* (OTU132), and *G*. *delicatula* (OTU266) seemed to increase at the same period. The species *G*. *delicatula* (OTU88) and the genus *Eucampia* sp. (OTU221) increased between September and December. It should be noted that different OTUs affiliated to the same species: *G*. *delicatula* (OTU88 and OTU266) and *G*. *flaccida* (OTU132 and OTU581) displayed different succession patterns. Several phytoplankton species, including the diatoms *Ditylum brightwellii* (OTU47) and *Thalassiosira hispida* (OTU17), the Mamiellophyceae *Bathycoccus prasinos* (OTU54) and *Micromonas* sp. (OTU66, OTU213), the Trebouxiophyceae *Chlorellales* sp. (OTU41), the Pyramimonadophyceae *Pycnococcus provasolii* (OTU110) and *Bolidophyceae* sp. (OTU168), increased between September and March. Generally, their RNA:DNA ratios were higher just before their peak of abundance and decreased drastically when their abundance became the highest (e.g. OTU16). Phytoplankton rRNA:rDNA ratio was highest in spring and summer (Fig 6B), with Chlorophytes displaying a median rRNA:rDNA ratio ~8.8 and ~6.5 in spring and summer, respectively, and Diatoms ~3.5 in spring (Fig 6B).

### *Phaeocystis globosa* life-cycle revealed by rRNA:rDNA ratio

**Fig 7:**
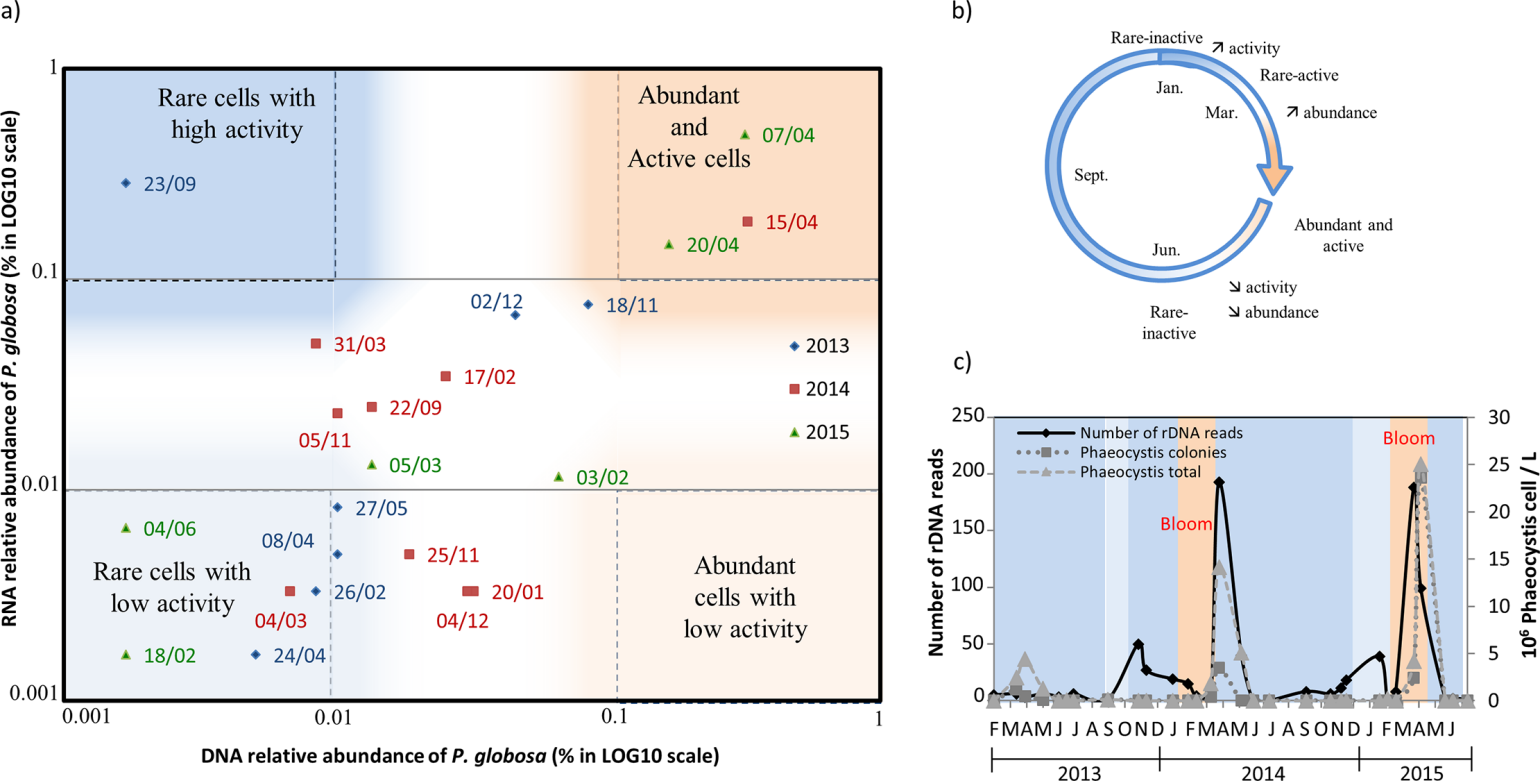
Relation between abundance (rDNA reads) and relative cell activity (rRNA reads) for *Phaeocystis globosa*. a) Relative rRNA and rDNA reads abundance corresponding to *P*. *globosa* between 2013 and 2015. Relative reads abundance for each sample were plotted on logarithmic scale. Considering abundance (rDNA-based data) and relative cell activity (rRNA-based data), four quadrants stand out: rare cells with low activity (DNA<0.01%, RNA<0.01%), rare-active cells (DNA<0.01%, RNA>0.1%), abundant-active cells (DNA>0.1%, RNA>0.1) and abundant cells with low activity (DNA>0.1%, RNA<0.01), respectively. Colors represent rDNA reads abundance (blue: rare, red: abundant), and shades represent rRNA reads abundance as proxy for relative cell activity (dark shade: active, light shade: low activity). b) Schematic representation of *P*. *globosa* annual pattern of abundance and activity in the EEC. c) Microscopic counts of *P*. *globosa* (dash light grey line: total number of *Phaeocystis* cells; dash dark grey line: number of *Phaeocystis* colony) and number of rDNA Illumina reads (black line).

From February to early-March, *P*. *globosa* was rare (rDNA reads abundance < 0.01% of all reads) and showed very low rRNA content (rRNA reads abundance < ~0.01% of all reads). In late March, *P*. *globosa* activity increased according to rRNA data but its abundance remained low according to rDNA data (rDNA < 0.01%, rRNA > 0.01%) (Fig 7A and 7B). Subsequently, it became abundant during its April bloom (rDNA>0.1%, rRNA>0.1%). Abundance (rDNA) began to decrease at the end of the bloom in mid-May, which was confirmed by decreasing number of *P*. *globosa* cells observed under microscope (Fig 7C). No rRNA reads were detected at the end of the senescence phase. From June to early February, *P*. *globosa* was again rare and display low relative cell activity (DNA<0.1%, RNA<0.1%, Fig 7A and 7B).

In 2013, the *P*. *globosa* bloom was observed with lower magnitude under microscope as compared to next two years, and was undetected with the Illumina approach (Fig 7C). Notwithstanding in November 2013 and February 2015, *P*. *globosa* rDNA read-increase was detected by Illumina sequencing suggesting a micro-bloom (Fig 7A and 7C). Interestingly, before the micro-bloom in November 2013, a significant increase of rRNA read abundance was observed in late September. This rRNA increase suggests higher relative cell activity in response to increase in nutrients (i.e NO_3_^-^+NO_2_^-^ 19.1 μM) and SST (14.7°C).

## Discussion

### Association between environmental variables and community structure

During this four-year study, environmental variables suggested three distinct seasonal periods: spring-early summer (bloom event of *P*. *globosa*), summer-early autumn, and late autumn-winter. As demonstrated by cluster analysis, microbial community structure (OTU) was generally associated with these three environmental periods. At the end of winter, the abundance of nitrate and high N:P and N:Si ratios triggered blooms of *P*. *globosa*, following by rises of colonial diatoms and heterotrophic dinoflagellates [41,42,43]. Microbial eukaryote diversity was at the lowest during *P*. *globosa* bloom period, but gradually increased to reach its highest in fall-winter. Symbionts and degraders accounted for more than one third of the OTU diversity in fall-winter [67]. Microbial eukaryote community structure and seasonal succession were similar between surveys and years for the most abundant taxonomic groups, representing the majority of reads (>90). Differences for rare groups (composed of less than ~10 OTUs) might result from natural diversity fluctuations in an open ecosystem. However, other biases due to random sampling, sequencing methods, PCR and sequencing errors might have also contributed to this disparity [68]. In addition, previous studies in the area have suggested that inter-taxa relations, rather than environmental variables, were the main drivers of microbial community structure and temporal succession [45,67]. These studies showed that environmental variables could explain only around 30% of microbial eukaryote succession [45], and few correlations were observed, through network analysis, between OTUs and environmental variables while correlations between microbes dominated the network [67].

### rDNA and rRNA-based microbial diversity survey

The diversity of planktonic eukaryotes was similar whether the target was rDNA or rRNA for the most abundant higher taxonomic groups (Fig 3B and 3E). In the cluster analysis, when considering the number of reads, one third of rDNA/rRNA pairs were grouped together, and 28 out of 30 pairs appeared in the same seasonal cluster. This result, although expected, suggests similar abundance estimation for most OTUs whether rRNA or rDNA is sequenced. However, weaker cluster definition and lower percentage of similarity is observed when considering the diversity of rare OTUs. This suggests that either reads of rare OTUs reached the detection limit of the sequencing method and therefore were not statistically significant, or that biases mentioned earlier hampered accurate detection for rare OTUs. In addition, we observed that RNA-based Bray-Curtis dissimilarity analysis has more consistent sample clustering according to environmental variables compared to DNA-based analysis (Fig 5C and 5D). This suggests that OTUs revealed by rRNA sequencing provides clearer seasonal representation of species succession and level of activity than rDNA-based OTU detection [3,26,69,70]. On the other hand, the DNA molecule is more stable than the RNA molecule and therefore allows thorough investigation for all present microbial species [70]. However, such stability could lead to several biases by including dead or dormant cells, and/or including DNA from the dissolved extracellular pools [71]. The rRNA-based surveys better depict microbial diversity, since they are less affected by ribosomal operon copy number between taxa [23] and reflect more accurately environmental changes due to their shorter lifetime compared to rDNA [25]. Overall, analyzing both rRNA and rDNA provide complementary information and should be considered for comprehensive understanding of ecosystems functioning.

### Relative activities of major microbial taxonomic and trophic groups

The comparison between rRNA- and rDNA-based surveys face several limitations including variations in gene copy number [66,72,73], cell size [65,66], and differences in life histories (e.g. dormant cells), and non-growth activities of certain species [29]. Nevertheless, this approach is nowadays generally accepted amongst environmental microbiologists to distinguish and quantify active species among present species, through calculation of the rRNA:rDNA ratio [12,22,23,74]. In our study, taxonomic groups in general displayed an average rRNA:rDNA ratio around 1:1, suggesting a linear relation between cell abundance (rDNA) and activities (rRNA). Two groups (i.e. Ciliophora and Choanoflagellida) appeared to have > 1 mean rRNA:rDNA ratio (slope > 1.25, Fig 6A). Therefore, these groups might be classified as RNA-prevalent, according to the definition of Massana et al., 2015. In addition, for these two groups, the negative b-value (intersection of the x- and y-axis) (Fig 6A) suggests that the relatively abundant OTUs were active, while the less abundant OTUs had lower than average relative activity. On the opposite, Fungi displayed < 1 mean rRNA/rDNA ratio (slope = 0.9 +/-0.07, Fig 6) and a positive b-value (b = +0.13 +/-0.004, Fig 6), that placed fungi in the DNA-prevalent category. According to these results, rare Ciliophora and Choanoflagellida probably have lower relative activity than the abundant ones, while rare Fungi display higher relative activity as compared with abundant fungi. However, care should be taken not to over-interpret the ecological relevance of prevalent (rDNA or rRNA) groups, and rRNA:rDNA regression. In fact, difference in reads’ abundance between rDNA- and RNA-based surveys might not necessarily result from variation in relative activities, but can also reflect differences in genome architecture between taxonomic groups, with RNA-prevalent groups having lower rDNA copy number [12]. It is worthy to underline that, while the overall rRNA:rDNA ratio was close to 1 for most taxonomic groups (ranging from 0.9 to 1.25), it was highly variable when considering individual OTUs (Fig 6A). Consequently, we suggest that the rRNA:rDNA ratio is more informative for individual OTUs, rather than whole taxonomic groups. Therefore, for each particular OTUs, significant changes in the rDNA:rRNA ratio may indicate ecological seasonal events, as for instance, it was observed for *P*. *globosa* (see the next section). Following RNA:rDNA ratio for individual OTUs, we often observed an increase of relative cells activity (rRNA reads) before increase of abundance (rDNA), suggesting the rRNA reads might be used as an early indicator of short-term microbial community dynamics in natural ecosystems. However, increased rRNA can be an indicator of any type of activities and not only those related to cell proliferation, thus it should always be interpreted with caution for "predicting" upcoming cell increase. Still, the rRNA:rDNA ratio provided insights into seasonal changes in activities of eukaryotic microbial taxa. We found that most groups (i.e Dinoflagellates, Ciliates, Chlorophytes, Haptophyta, Diatoms, and Fungi) had OTUs displaying their highest activity in spring and summer (Fig 6B). A similar observation was made at a coastal station in the eastern North Pacific where the majority of protistan groups had significant higher rRNA:rDNA ratios in April [26]. However, the relative activity across OTUs is very different, as shown by long whiskers in the rRNA:rDNA boxplot diagram (Fig 6B). Therefore, thorough investigation of each individual OTU's variation in rRNA:rDNA ratio, and their association with environmental parameters, is necessary to understand species succession and seasonal response in ecosystems. Such analysis performed for *P*. *globosa* demonstrated its relevance (next section). Finally, while rRNA:rDNA ratio may reveal cells activity, information about which kind of activity is expressed remains unknown. In future studies, *in situ* metatranscriptomic analysis on time-series will help to better understand how species adapt their physiology and metabolism in response to changing environmental conditions, how they interact with other organisms, and what are their roles and functions in ecosystems.

### Phases of *Phaeocystis globosa* life-cycle emerge from the rRNA:rDNA ratio

*Phaeocystis* has a complex life-cycle alternating between solitary cells and colonies, and is capable of forming massive, quasi-monospecific blooms in naturally or anthropogenetically induced nitrogen rich areas [75]. The present data based on high throughput sequencing of rDNA and rRNA detected the bloom of *P*. *globosa* but also showed that *P*. *globosa* is always present in the ecosystem throughout the entire year. Yet, while rRNA:rDNA ratio used to evaluate cells activity was informative, it appeared that simply calculating this ratio only gave partial results for activity as rRNA and rDNA sequences often increase proportionally (Fig 6). In order to determine the general trend for rRNA and rDNA increase or decrease during time-series, graphical relation between relative amount of rRNA and rDNA reads was constructed (Fig 7A). To note that determining the level of relative reads abundance to differentiate rare / abundant and active / inactive organisms is not straightforward, as it is affected by nucleic acids extraction efficiency, primer specificities, and by variation of rDNA gene copy number per cell. Our choice was to select levels of abundance and activity presented on Fig 7A considering that cells were rare when their relative rDNA reads abundance on total rDNA reads was below 0.01% and abundant when the relative rDNA reads number was above 0.1% [10]. The same thresholds were considered for rRNA in order to differentiate high versus low activity cells. These levels are only indicative and can be refined in the future by additional data. This approach allowed delimiting four regions corresponding to: (1) rare cells with low activity, (2) rare and active cells, (3) abundant cells with low activity, and (4) abundant and active cells (Fig 7A). Samples that did not belong to any of the above categories could be considered in transition state between phases. Overall, the levels used here to delimit abundance and activity unveiled quite well the bloom of *Phaeocystis* in early- / mid-April, but more interestingly, the increase of cells activity before the bloom and activity decrease after the bloom. According to these results, rRNA reads number could be an indicator of an upcoming bloom, provided that the sampling is frequent enough. In 2013, the bloom of *P*. *globosa* was not detected in the illumina dataset but was detected in the pyrosequencing dataset [45,46]. This could be due to the degradation of ribosomal DNA prohibiting its detection, combined with the weaker bloom observed that year under microscope. Finally, a fall-winter increase of *P*. *globosa* was shown by molecular analysis (rDNA) at the end of 2013 and, to a lower extent, in early 2015. In end-September 2013, an increase of rRNA sequences, suggesting higher cell activities, preceded the increase of abundance (rDNA sequences) three weeks later, in mid-November 2013. Data point characteristics for the increase are positioned within transition phases (e.g November 18^th^ and December 2^nd^ 2013, Fig 7A). This is not surprising considering the lower magnitude of this bloom, as compared with the main mid-April bloom. It also highlights the difficulty to clearly define boundaries between rare/abundant and active/inactive cells.

Concluding, this study showed consistent detection of microbial eukaryote diversity whether targeting rDNA or rRNA, and the possibility to use rRNA:rDNA relative read abundance as a "life-event" indicator. Generalizing this approach on individual OTUs would contribute into improving our understanding of microbial community succession and of the underlying factors structuring the community.

## Supporting information

S1 Table Physical-chemical parameters and chlorophyll a (Chl. a) from March 2011 to July 2015 at the SOMLIT station in the eastern English Channel (SOMLIT network, http://somlit.epoc.u-bordeaux1.fr/fr/). Kd: diffuse attenuation coefficient, PAR: photosynthetically active radiation, T: temperature, S: salinity, O2: Oxygen, pH: potential hydrogen, NO_3_^-^: nitrate, NO_2_^-^: nitrite, PO_4_^3-^: phosphate, SiOH4: silicate, POC: particulate organic carbon, PON: particulate organic nitrogen, SPM: suspended particular matter, Chl. a: chlorophyll a, and N/P: nitrate/phosphate ratio.
(DOCX)
