# Supplementary material for: Diversity and potential activity patterns of planktonic eukaryotic microbes in a mesoeutrophic coastal area (eastern English Channel)
Source: PLoS One. 2018 May 10;13(5):e0196987. doi: 10.1371/journal.pone.0196987 (PMC5944946; doi:10.1371/journal.pone.0196987)
Supplement: S1 Table — Kd: diffuse attenuation coefficient, PAR: photosynthetically active radiation, T: temperature, S: salinity, O2: Oxygen, pH: potential hydrogen, NO3-: nitrate, NO2-: nitrite, PO43-: phosphate, SiOH4: silicate, POC: particulate organic carbon, PON: particulate organic nitrogen, SPM: suspended particular matter, Chl. a: chlorophyll a, and N/P: nitrate/phosphate ratio. (DOCX) [file pone.0196987.s001.docx]

**S1 Table. Physical-chemical parameters and Chlorophyll *a* (Chl *a*) from March 2011 to July 2015 at the SOMLIT station in the eastern English Channel (SOMLIT network,** [**http://somlit.epoc.u-bordeaux1.fr/fr/**](http://somlit.epoc.u-bordeaux1.fr/fr/)**).**

| Dates | Kd (m^-1^) | PAR  (E m^-2^ d^-1^) | T (°C) | Salinity | O_2_ (mg L^-1^) | pH | NO_3_+NO_2_  (μM) | PO_4_  (μM) | SiOH_4_  (μM) | POC  (µgC L^-1^) | PON  (µgN L^-1^) | SPM  (mg L^-1^) | Chl *a*  (μg L^-1^) | | N/P | |
| --- | --- | --- | --- | --- | --- | --- | --- | --- | --- | --- | --- | --- | --- | --- | --- | --- |
| 07/03/2011 | 0,31 | 19,28 | 6,80 | 34,20 | 7,50 | 8,26 | 4,32 | 0,10 | 0,25 | 219,72 | 38,67 | 4,36 | 5,80 | 43,20 | |  |
| 21/03/2011 | 0,17 | 19,03 | 7,60 | 33,90 | 8,10 | 8,37 | 7,10 | 0,10 | 0,10 | 207,06 | 45,17 | 2,91 | 4,50 | 71,00 | |  |
| 04/04/2011 | 0,43 | 17,85 | 9,00 | 34,40 | 7,60 | 8,40 | 1,57 | 0,07 | 0,17 | 300,77 | 45,63 | 3,29 | 9,90 | 22,40 | |  |
| 18/04/2011 | 0,36 | 25,76 | 10,50 | 34,20 | 8,10 | 8,42 | 0,20 | 0,34 | 2,37 | 394,48 | 46,09 | 7,04 | 11,70 | 0,59 | |  |
| 04/05/2011 | 0,30 | 37,02 | 11,80 | 34,50 | 6,50 | 8,30 | 0,10 | 0,13 | 0,43 | 355,94 | 60,26 | 6,29 | 1,90 | 0,77 | |  |
| 06/06/2011 | 0,21 | 38,66 | 15,20 | 34,50 | 5,50 | 8,24 | 0,31 | 0,30 | 1,57 | 278,82 | 58,62 | 3,69 | 1,30 | 1,00 | |  |
| 15/06/2011 | 0,28 | 34,31 | 15,50 | 34,70 | 5,60 | 8,22 | 0,25 | 0,26 | 1,29 | 297,75 | 51,32 | 1,52 | 0,90 | 1,00 | |  |
| 04/07/2011 | 0,22 | 51,96 | 17,70 | 34,50 | 5,90 | 8,22 | 0,34 | 0,05 | 1,66 | 237,84 | 35,61 | 2,28 | 1,30 | 6,80 | |  |
| 27/09/2011 | 0,21 | 23,98 | 17,00 | 34,50 | 5,60 | 8,20 | 3,12 | 0,16 | 2,88 | 178,97 | 35,01 | 3,13 | 2,20 | 19,50 | |  |
| 25/10/2011 | 0,23 | 17,84 | 14,20 | 34,20 | 5,80 | 8,22 | 3,65 | 0,12 | 0,87 | 231,95 | 50,59 | 3,21 | 6,30 | 30,40 | |  |
| 09/11/2011 | 0,21 | 4,43 | 14,20 | 34,60 | 5,50 | 8,16 | 8,67 | 0,41 | 1,82 | 152,37 | 19,11 | 4,16 | 0,70 | 21,10 | |  |
| 23/11/2011 | 0,18 | 10,12 | 12,10 | 34,50 | 5,90 | 8,11 | 11,20 | 0,43 | 0,35 | 153,49 | 22,20 | 1,59 | 0,40 | 26,00 | |  |
| 24/01/2012 | 0,52 | 3,62 | 8,10 | 33,90 | 6,40 | 8,18 | 15,10 | 0,75 | 6,21 | 154,60 | 25,30 | 10,31 | 1,00 | 20,10 | |  |
| 20/03/2012 | 0,55 | 19,68 | 7,40 | 34,10 | 8,20 | 8,34 | 4,53 | 0,59 | 0,51 | 425,70 | 25,92 | 1,41 | 1,90 | 7,70 | |  |
| 05/04/2012 | 0,67 | 19,80 | 8,50 | 34,50 | 8,20 | 8,38 | 0,20 | 1,06 | 3,10 | 576,48 | 5,33 | 2,76 | 4,50 | 0,19 | |  |
| 09/05/2012 | 0,23 | 20,62 | 10,90 | 34,70 | 6,70 | 8,33 | 0,14 | 1,20 | 1,07 | 328,81 | 36,22 | 1,86 | 3,40 | 0,12 | |  |
| 05/06/2012 | 0,20 | 27,64 | 14,30 | 34,60 | 6,40 | 8,30 | 0,11 | 0,10 | 0,83 | 520,67 | 35,74 | 0,96 | 2,10 | 1,10 | |  |
| 21/06/2012 | 0,19 | 47,41 | 15,80 | 34,40 | 5,70 | 8,24 | 1,02 | 0,42 | 0,93 | 341,90 | 41,85 | 0,10 | 0,50 | 2,43 | |  |
| 04/07/2012 | 0,32 | 40,65 | 17,00 | 34,00 | 6,20 | 8,22 | 0,61 | 0,21 | 0,37 | 494,52 | 56,78 | 1,16 | 8,00 | 2,90 | |  |
| 23/07/2012 | 0,36 | 38,93 | 17,60 | 33,50 | 5,90 | 8,23 | 0,18 | 0,22 | 0,96 | 481,78 | 61,56 | 2,09 | 7,90 | 0,82 | |  |
| 03/09/2012 | 0,28 | 25,74 | 18,50 | 34,20 | 5,40 | 8,14 | 0,10 | 0,22 | 1,74 | 268,62 | 37,72 | 1,27 | 2,80 | 0,45 | |  |
| 30/10/2012 | 0,50 | 7,84 | 12,80 | 33,30 | 5,70 | 8,12 | 12,10 | 0,42 | 7,70 | 230,00 | 50,00 | 7,86 | 1,00 | 28,80 | |  |
| 13/11/2012 | 0,61 | 5,91 | 11,80 | 34,20 | 5,90 | 8,10 | 11,10 | 0,45 | 5,30 | 150,00 | 18,00 | 3,01 | 1,80 | 24,70 | |  |
| 11/02/2013 | 0,45 | 3,80 | 6,50 | 33,80 | 6,80 | 8,19 | 9,23 | 0,43 | 2,92 | 260,00 | 44,85 | 3,17 | 3,60 | 21,45 | |  |
| 26/02/2013 | 0,49 | 7,46 | 5,87 | 34,30 | 7,08 | 8,22 | 7,35 | 0,41 | 1,14 | 370,43 | 71,69 | 3,07 | 9,95 | 17,93 | |  |
| 26/03/2013 | 0,27 | 16,32 | 5,40 | 34,40 | 7,30 | 8,30 | 3,15 | 0,15 | 0,28 | 391,24 | 75,97 | 2,14 | 3,20 | 21,00 | |  |
| 08/04/2013 | 0,49 | 21,41 | 5,44 | 34,41 | 7,20 | 8,29 | 2,89 | 0,25 | 0,01 | 412,04 | 80,24 | 6,16 | 4,42 | 11,56 | |  |
| 24/04/2013 | 0,30 | 26,30 | 7,86 | 34,41 | 7,60 | 8,37 | 0,60 | 0,09 | 0,01 | 584,20 | 83,56 | 2,70 | 8,33 | 6,67 | |  |
| 27/05/2013 | 0,23 | 40,31 | 11,26 | 34,10 | 6,57 | 8,38 | 2,10 | 0,04 | 0,01 | 271,12 | 48,88 | 1,18 | 1,94 | 52,50 | |  |
| 10/06/2013 | 0,64 | 32,72 | 11,90 | 34,30 | 6,40 | 8,43 | 0,29 | 0,89 | 1,12 | 756,00 | 72,24 | 2,16 | 1,20 | 0,33 | |  |
| 25/06/2013 | 0,66 | 21,52 | 14,98 | 33,64 | 6,63 | 8,49 | 0,21 | 0,18 | 0,01 | 1242,80 | 95,60 | 3,14 | 14,19 | 1,17 | |  |
| 09/07/2013 | 0,29 | 42,47 | 15,71 | 34,16 | 5,96 | 8,39 | 0,36 | 0,12 | 0,10 | 713,08 | 68,15 | 1,90 | 5,21 | 3,00 | |  |
| 22/07/2013 | 0,23 | 46,11 | 17,31 | 34,18 | 5,31 | 8,28 | 0,28 | 0,06 | 0,79 | 451,24 | 50,37 | 0,65 | 2,07 | 4,67 | |  |
| 23/09/2013 | 0,29 | 21,61 | 17,01 | 33,81 | 5,10 | 8,22 | 1,09 | 0,22 | 1,88 | 189,39 | 32,58 | 1,77 | 1,81 | 4,95 | |  |
| 18/11/2013 | 0,33 | 4,75 | 12,39 | 33,39 | 5,86 | 8,17 | 19,10 | 0,66 | 13,60 | 154,68 | 39,95 | 5,42 | 2,16 | 28,94 | |  |
| 02/12/2013 | 0,36 | 4,44 | 10,20 | 33,73 | 6,09 | 8,17 | 24,10 | 0,59 | 12,90 | 64,78 | 28,95 | 6,39 | 1,51 | 40,85 | |  |
| 20/01/2014 | 0,19 | 4,82 | 8,69 | 33,11 | 6,36 | 8,21 | 26,12 | 0,61 | 12,32 | 149,57 | 24,06 | 1,67 | 1,54 | 42,82 | |  |
| 17/02/2014 | 0,46 | 9,19 | 7,89 | 33,28 | 6,47 | 8,01 | 26,71 | 0,56 | 10,66 | 207,58 | 44,25 | 6,15 | 2,12 | 47,70 | |  |
| 04/03/2014 | 0,35 | 13,27 | 8,42 | 33,84 | 6,51 | 8,05 | 18,43 | 0,46 | 6,44 | 297,24 | 38,43 | 5,27 | 2,95 | 40,07 | |  |
| 31/03/2014 | 0,66 | 19,14 | 9,62 | 34,06 | 7,76 | 8,30 | 4,30 | 0,02 | 0,26 | 440,12 | 52,98 | 1,72 | 5,01 | 215,00 | |  |
| 15/04/2014 | 0,68 | 28,95 | 10,93 | 34,39 | 7,46 | 8,33 | 0,56 | 0,36 | 0,51 | 820,24 | 140,32 | 3,56 | 5,91 | 1,56 | |  |
| 26/05/2014 | 0,19 | 33,99 | 14,16 | 34,29 | 5,91 | 8,19 | 0,07 | 0,01 | 1,80 | 330,65 | 84,45 | 1,20 | 1,34 | 7,00 | |  |
| 17/06/2014 | 0,26 | 38,25 | 15,81 | 34,42 | 5,48 | 8,15 | 0,27 | 0,06 | 1,69 | 333,08 | 89,16 | 1,47 | 3,22 | 4,50 | |  |
| 16/07/2014 | 0,29 | 38,65 | 18,42 | 34,18 | 5,35 | 8,22 | 0,09 | 0,03 | 0,70 | 241,37 | 48,01 | 1,82 | 2,74 | 3,00 | |  |
| 22/09/2014 | 0,31 | 22,45 | 18,47 | 34,35 | 4,92 | 8,10 | 1,10 | 0,16 | 3,25 | 228,92 | 50,88 | 2,01 | 2,89 | 6,88 | |  |
| 05/11/2014 | 0,52 | 5,26 | 14,65 | 34,07 | 5,50 | 8,13 | 10,54 | 0,30 | 2,64 | 231,68 | 59,68 | 3,73 | 7,66 | 35,13 | |  |
| 25/11/2014 | 0,44 | 5,11 | 12,52 | 34,21 | 5,73 | 8,12 | 12,85 | 0,40 | 4,87 | 192,80 | 55,19 | 3,91 | 2,59 | 32,13 | |  |
| 04/12/2014 | 0,68 | 2,28 | 11,69 | 34,21 | 5,71 | 8,12 | 13,80 | 0,47 | 4,94 | 198,40 | 46,25 | 14,22 | 2,46 | 29,36 | |  |
| 03/02/2015 | 0,29 | 6,78 | 7,18 | 34,01 | 6,46 | 8,09 | 16,88 | 0,37 | 8,58 | 102,30 | 33,27 | 15,43 | 1,60 | 45,62 | |  |
| 18/02/2015 | 0,64 | 11,53 | 6,44 | 33,73 | 7,41 | 8,14 | 13,74 | 0,32 | 4,64 | 345,00 | 65,80 | 19,96 | 2,85 | 42,94 | |  |
| 05/03/2015 | 0,61 | 15,33 | 7,06 | 33,17 | 7,65 | 8,26 | 15,80 | 0,14 | 1,16 | 369,90 | 43,80 | 18,95 | 13,13 | 112,86 | |  |
| 07/04/2015 | 0,73 | 17,28 | 8,98 | 33,72 | 7,35 | 8,30 | 7,47 | 0,02 | 0,41 | 288,40 | 55,00 | 9,03 | 8,12 | 373,50 | |  |
| 20/04/2015 | 0,57 | 30,54 | 10,31 | 33,96 | 8,18 | 8,38 | 1,35 | 0,08 | 0,38 | 928,84 | 180,53 | 4,97 | 9,14 | 16,88 | |  |
| 04/06/2015 | 0,23 | 36,02 | 14,58 | 34,01 | 6,12 | 8,24 | 1,19 | 0,21 | 0,29 | 1125,80 | 103,80 | 3,58 | 4,64 | 5,67 | |  |
| 18/06/2015 | 0,27 | 37,37 | 16,09 | 33,99 | 5,74 | 8,26 | 1,82 | 0,03 | 0,28 | 522,90 | 98,90 | 32,93 | 2,85 | 60,67 | |  |
| 15/07/2015 | 0,25 | 28,39 | 17,89 | 34,19 | 5,07 | 8,09 | 0,19 | 0,06 | 0,89 | 355,60 | 75,90 | 22,62 | 1,33 | 3,17 | |  |

Kd: diffuse attenuation coefficient, PAR: photosynthetically active radiation, T: temperature, S: salinity, O_2_: Oxygen, pH: potential hydrogen, NO_3_: nitrate, NO_2_: nitrite, PO_4_: phosphate, SiOH4: silicate, POC: particulate organic carbon, PON: particulate organic nitrogen, SPM: suspended particular matter, Chl: chlorophyll a, and N/P: nitrate/phosphate ratio.
